# Hypothalamic Integration of Metabolic, Endocrine, and Circadian Signals in Fish: Involvement in the Control of Food Intake

**DOI:** 10.3389/fnins.2017.00354

**Published:** 2017-06-26

**Authors:** María J. Delgado, José M. Cerdá-Reverter, José L. Soengas

**Affiliations:** ^1^Departamento de Fisiología (Fisiología Animal II), Facultad de Biología, Universidad Complutense de MadridMadrid, Spain; ^2^Departamento de Fisiología de Peces y Biotecnología, Instituto de Acuicultura Torre de la Sal, Consejo Superior de Investigaciones CientíficasCastellón, Spain; ^3^Laboratorio de Fisioloxía Animal, Departamento de Bioloxía Funcional e Ciencias da Saúde, Facultade de Bioloxía, Universidade de VigoVigo, Spain

**Keywords:** food intake, fish, hypothalamus, review, nutrient sensing, circadian rhythm, leptin, ghrelin

## Abstract

The regulation of food intake in fish is a complex process carried out through several different mechanisms in the central nervous system (CNS) with hypothalamus being the main regulatory center. As in mammals, a complex hypothalamic circuit including two populations of neurons: one co-expressing neuropeptide Y (NPY) and Agouti-related peptide (AgRP) and the second one population co-expressing pro-opiomelanocortin (POMC) and cocaine- and amphetamine-regulated transcript (CART) is involved in the integration of information relating to food intake control. The production and release of these peptides control food intake, and the production results from the integration of information of different nature such as levels of nutrients and hormones as well as circadian signals. The present review summarizes the knowledge and recent findings about the presence and functioning of these mechanisms in fish and their differences vs. the known mammalian model.

## Introduction

Food intake is regulated through positive and negative loops acting at different locations and at different times (Langhans and Scharrer, 1992; Langhans, 1999). The positive loop (starting of food intake) results from the relationship among prior experience with nutrient availability, status of the animal and sensory qualities of the food. The negative loop relates to metabolic and gastrointestinal inputs displaying changes prior and after absorption (Langhans, 1999). Three regulatory levels have been suggested following this model: (i) short-term regulatory factors: those influenced by the size of a single meal, (ii) mid-term regulatory factors: those operating through several days; and (iii) long-term regulatory factors: those operating through longer time periods (weeks, months, and years) reflecting energy balance of the animal. These levels of regulation create a feedback loop that continuously modulate the immediate signal input from sense organs and internal sensors, which together with prior learning integrate information from the energy reserves of the body. The control determining the feeding behavior and food intake is elicited by the central nervous system (CNS) through various pathways, and hypothalamus is the main center involved in such regulation (Schwartz et al., 2000; Berthoud, 2002).

**Figure 1:**
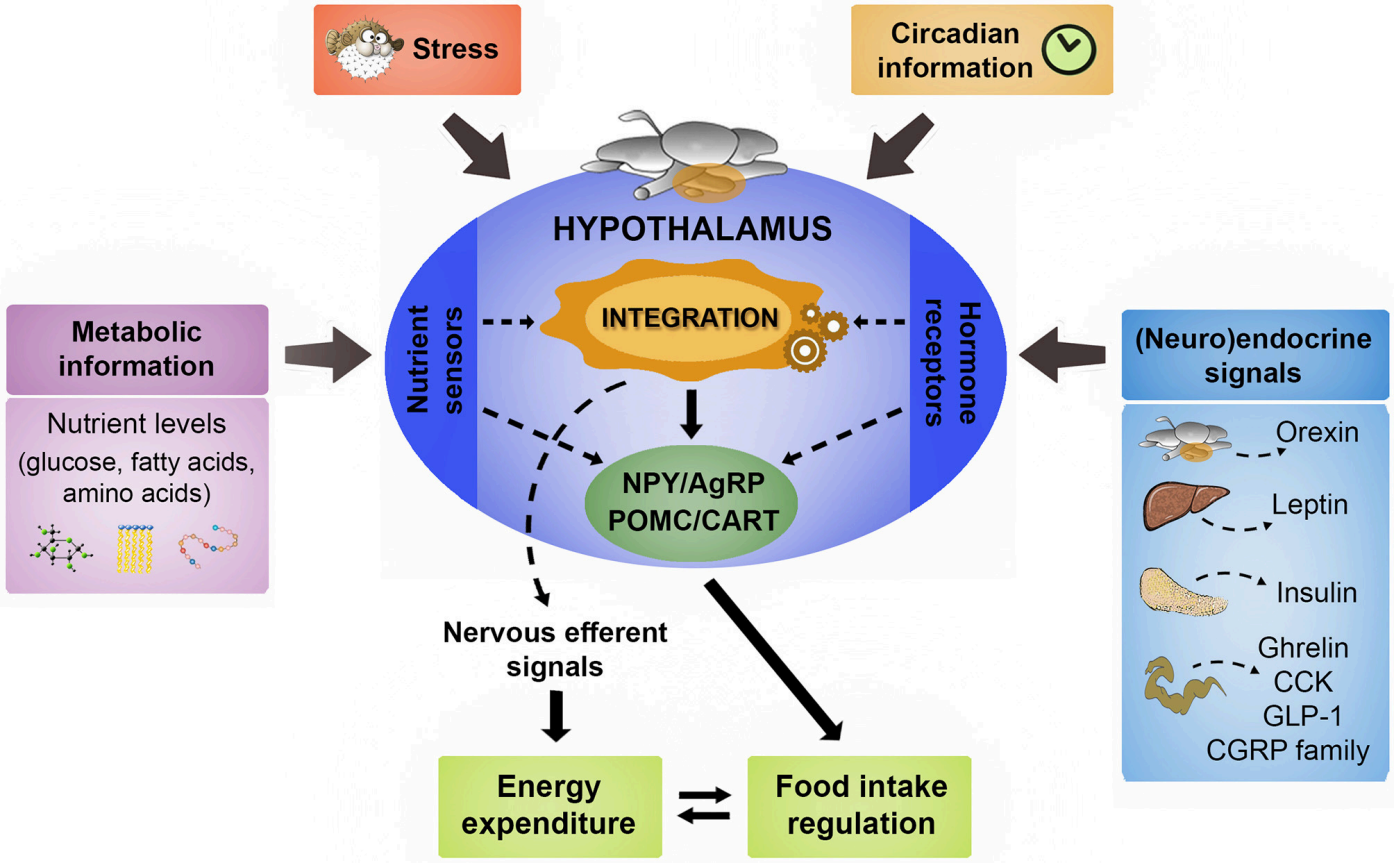
Schematic representation illustrating the main signaling elements involved in the hypothalamic control of food intake in fish. A comprehensive set of central and peripheral neuroendocrine messengers in a coordinated way with the metabolic information provided by nutrient levels constitute the main signaling that regulates hypothalamic neurocircuits involved in the regulation of food intake. The detailed description of such main functional neuropeptidergic circuits is shown in the text. The hypothalamus also integrates the stress response in the generation of the adaptive changes in food intake and energy expenditure according to the exposure to stressful conditions. Finally, the role of hypothalamus in the internal coordination of circadian rhythmicity related to feeding behavior is also described in the text. AgRP, agouti-related peptide; CART, cocaine- and amphetamine-related transcript; CCK, cholecystokinin; CGRP, calcitonin gene-related peptide; GLP-1, glucagon-like peptide 1; POMC, pro-opiomelanocortin; NPY, neuropeptide Y.

In fish is difficult to establish which factors act in the mid- or long-term, since most available data come from short-term studies. The basic mechanisms involved in regulation of food intake in fish appear to be similar to those of mammals with differences implying the existence of specific mechanisms in fish (Kulczykowska and Sánchez-Vázquez, 2010; Hoskins and Volkoff, 2012; Volkoff, 2016). In the following sections, we review the existing knowledge about hypothalamic integration in fish of metabolic, endocrine, and circadian information to elicit a coordinated feeding response, as summarized in Figure 1.

## Hypothalamic neuropeptides involved in the control of food intake

The integration of information involved in the control of food intake takes place in mammals through a circuit mainly localized in hypothalamic areas including arcuate (ARC), ventromedial, paraventricular, and lateral hypothalamus (Berthoud and Morrison, 2008; Zheng and Berthoud, 2008). These circuits include two populations of neurons (Mobbs et al., 2005; Blouet and Schwartz, 2010; Waterson and Horvath, 2015). The first population co-expresses neuropeptide Y (NPY) and agouti-related peptide (AgRP) and the second population co-expresses pro-opiomelanocortin (POMC) and cocaine- and amphetamine-regulated transcript (CART). Moreover, these two populations of neurons inhibit each other resulting in signaling to other higher-order neurons. The production and release of these peptides control food intake, and this occurs through integration of signals of metabolic, endocrine, and circadian nature. Other peptides such as orexins are involved in food intake modulation but they are not involved in this main integrative core.

Avaliable studies in fish (mostly through asssessment of mRNA abundance) demonstrated expression of these neuropeptides in hypothalamus. Neurons expressing NPY, AgRP, CART, and POMC in fish respond to energy challenge suggesting that the main hypothalamic pathways coordinating a response to regulate energy homeostasis are conserved throughout vertebrate evolution (Volkoff et al., 2005).

### CART/POMC

Melanocortins integrate a key system in the regulation of food intake in vertebrates. These peptides, exhibiting melanotropic (melanin stimulation) and corticotropic (corticosteroid stimulation) activity encoded in the common precursor POMC. Two discrete groups of neurons in ARC and the caudal region of the nucleus of the tractus solitarius of the medulla produce POMC, mainly processed into α-melanocyte-stimulating hormone (α-MSH) and β-endorphin. Melanocortin signaling is transduced by five different G-coupled receptors (MC1R-MC5R) but only two are conspicuously expressed in the mammalian brain, i.e., MC3R and MC4R. Central activation of these receptors appears to mediate melanocortin effects on energy balance since both MC3R knockout rat and MC4R knockout mice display severe alterations in energy homeostasis (obesity, increased food intake and linear growth in MC4R; hypophagia, increased fat mass and food efficiency but reduced lean mass in MC3R). Accordingly, central administration of MC3/4Rs agonists results in a dose-dependent reduction in food intake in mice but MC4R-deficient mice do not respond to anorectic effects of agonists, suggesting that α-MSH inhibits feeding primarily by activating MC4R (reviewed by Anderson et al., 2016).

Several studies demonstrated that melanocortin system is a key point in the regulation of energy balance in fish (Cerdá-Reverter et al., 2011). Thus, *in situ* hybridization studies demonstrated the presence of POMC neurons in hypothalamus (Cerdá-Reverter et al., 2003a) but not in the vagal lobe (homolog of the nucleus of the tractus solitarius in mammalian brain). However, more sensitive techniques (quantitative PCR) also reported POMC-A1 expression in the hindbrain (Conde-Sieira et al., 2010b). Immunohistochemical techniques further demonstrated the presence of both α-MSH and β-endorphin in the hypothalamus of several fish species, including zebrafish and tilapia (Forlano and Cone, 2007; Chabbi and Ganesh, 2016) thus suggesting that POMC is mainly processed to these peptides. However, ACTH is also present in the preoptic area of carp (Metz et al., 1994) further suggesting an alternative POMC processing in the fish brain.

Intracerebroventricular (ICV) administration of α-MSH or α-MSH agonists inhibit food intake in a dose-dependent manner in goldfish (Cerdá-Reverter et al., 2003b) and rainbow trout (Schjolden et al., 2009), whereas administration of MC4R antagonists stimulates food intake in satiated animals (Cerdá-Reverter et al., 2003b). POMC-A1 and POMC-C levels increased post-feeding in medaka (Chisada et al., 2014), rainbow trout (Gong and Björnsson, 2014), and Atlantic halibut (Gomes et al., 2015), respectively. In contrast to that expected for a negative regulator of energy balance, POMC hypothalamic levels remained unchanged after 7 progressive fasting days in goldfish (Cerdá-Reverter et al., 2003a), but POMC-A1 mRNA decreased 50% after 28 fasting days in rainbow trout (Leder and Silverstein, 2006). Surprisingly, another study demonstrated that hypothalamic POMC A1, A2 and B levels increased after 118 fasting days in the latter species (Jørgensen et al., 2016). The lack of a consistent POMC regulation by energy balance also occurred in mammalian species. Thus, sheep with about 40% of total body-weight loss did not exhibit changes in POMC mRNA levels (Henry et al., 2001). A similar situation occurred in ewes that lost about 30% body fat (Henry et al., 2000). The constrasting effects of POMC-encoded peptides could explain the paradoxical absence of POMC regulation by energy balance. Thus, central injections of β-endorphin, the C-terminal peptide of POMC, stimulate appetite in the goldfish (De Pedro et al., 1995). It is then plausible that the effects of nutritional status might regulate post-transcriptional POMC-derived peptide levels by selective regulation of prohormone convertases. Under negative energy balance situations, when animals display an enhanced feeding drive, POMC might be processed into β-endorphin. In contrast, a positive energy balance may preferentially drive the processing of POMC into production of the food intake inhibitor α-MSH.

The melanocortin activity may be also modulated by receptor signaling. Accordingly, energy balance, in the absence of an agonist regulation, could up/down regulate the neuronal receptor density in feeding-related areas of the CNS. Studies in the sea bass suggest that hypothalamic MC4R expression remains unchanged after long-term fasting precluding this regulatory pathway (Sánchez et al., 2009). Alternatively, the melanocortin system also exhibits endogenous antagonists competing by binding and activation of the melanocortin receptors. AgRP1 is consistently upregulated by fasting in the hypothalamus of all tested vertebrates including zebrafish (Song et al., 2003), goldfish (Cerdá-Reverter and Peter, 2003), Atlantic salmon (Valen et al., 2011), and sea bass (Agulleiro et al., 2014). It is therefore conceivable that AgRP1 binding to central MCRs might regulate central melanocortinergic activity whereas POMC expression remains constant as a constitutive inhibitor.

In mammals, ARC POMC neurons also produce CART but hypothalamus is not the only brain area producing this neuropeptide (Elias et al., 1998). CART was isolated form ovine hypothalamus (Spiess et al., 1981) and its expression increased in the rat *striatum* after the administration of drugs such as cocaine and amphetamine (Douglass et al., 1995). Subsequent experiments revealed that CART neurons are anatomical targets for systemic leptin to induce anorexia (Kristensen et al., 1998). In fish, CART mRNA was characterized in several species (Subhedar et al., 2014) but immunohistochemical localization of the CART peptide in brain was only studied in catfish by using antibodies against rat CART (Singru et al., 2007) or by *in situ* hybridization in zebrafish (Nishio et al., 2012; Akash et al., 2014). CART mRNA abundance decreased with food deprivation in cod (Kehoe and Volkoff, 2007), goldfish (Volkoff and Peter, 2001a), and Atlantic salmon (Murashita et al., 2009), and increased with re-feeding in channel catfish (Kobayashi et al., 2008) whereas post-prandial changes occurred in channel catfish (Peterson et al., 2012), goldfish (Volkoff and Peter, 2001a), and dourado (Volkoff et al., 2016). As many other genes, CART is duplicated in the teleost genome and four different genes (CART1-4 or CART1, 2a, 2b, 4) encoding CART peptides are reported in zebrafish (Nishio et al., 2012) or up to seven genes in Senegalese sole (Bonacic et al., 2015). All of them are expressed in zebrafish CNS but only CART2 and 4 are produced in the hypothalamus (Akash et al., 2014). Unfortunately, CART/POMC colocalization in the neurons of the tuberal hypothalamus has not been assessed in fish yet. Both CART2 and CART4 are downregulated by fasting in ventrocaudal telencephalon (CART2) and ventral hypothalamus (CART2 and CART4) (Akash et al., 2014), as expected for a negative regulator of the energy balance and thus further suggesting their involvement in the regulation of energy balance in fish. Accordingly, ICV administration of CART peptides inhibit food intake in goldfish (Volkoff and Peter, 2000).

### AgRP/NPY

Atypically, melanocortin signaling is not exclusively regulated by the binding of endogenous agonists (see above), since naturally occurring antagonists, agouti-signaling protein (ASIP) and AgRP, compete with melanocortin peptides by binding to MCRs. ASIP and AgRP molecules exhibit a cysteine-rich C-terminal domain essential for the structural and biological properties of the peptide. A basic domain and a proline-rich area precede the cysteine knot in ASIP; AgRP sequences lacks both regions but, in contrast, exhibits a processing site prior to the cysteine domain where the propeptide is cleaved (reviewed by Cerdá-Reverter et al., 2011). In mammalian species, ASIP produced in the ventral skin regulates pigment pattern and binds MC1R and MC4R with similar affinity (Cerdá-Reverter et al., 2005). In contrast, AgRP is mainly expressed in the same NPY-producing neurons in the ARC. In fact, 95% of NPY neurons co-express AgRP (Hahn et al., 1998). AgRP/NPY neurons also produce GABA (Horvath et al., 1997) and regulate the activity of hypothalamic POMC/CART neurons. In addition, the GABAergic innervation from AgRP/NPY neurons to the parabrachial nucleus in the brainstem is able to regulate feeding behavior (Wu et al., 2009). Hypothalamic AgRP sharply increases with fasting, and overexpression in transgenic mice induces overfeeding and obesity but also enhances linear growth (Ollmann et al., 1997). The selective ablation of NPY/AgRP neurons induces a rapid decrease in food intake leading to starvation (Luquet et al., 2005).

Fish exhibit two copies of AgRP named AgRP1 and AgRP2. Few studies to date have precisely localized AgRP1 mRNA in brain regions of teleost fish (Cerdá-Reverter and Peter, 2003; Song and Cone, 2007) whereas others reported AgRP-ir in fish brain (Forlano and Cone, 2007; Agulleiro et al., 2014). All studies identified AgRP1 production within the posterior region of the ventral hypothalamus of the goldfish, zebrafish and sea bass. In the zebrafish, AgRP1 and α-MSH projections strikingly match the nuclei that express MC4R (Cerdá-Reverter et al., 2003b) and MC5R mRNA (Cerdá-Reverter et al., 2003c) in the goldfish. In fact, binding studies with zebrafish receptors demonstrated that AgRP1 acts as a competitive antagonist at MC3R, MC4R, and MC5R, all of them expressed in the brain (Song and Cone, 2007). Pharmacological studies in sea bass have shown that AgRP1 works as an inverse agonist at constitutively activated MC4R (Sánchez et al., 2009). In addition, ASIP1 overexpression working at central MC4R in transgenic zebrafish model results in enhanced feeding, feed efficiency, weight and linear growth but not in obesity (Guillot et al., 2016), in a similar way to the AgRP1 transgenic zebrafish (Song and Cone, 2007). Studies in zebrafish using first developmental stages demonstrated that AgRP1 knockdown results in reduced growth suggesting that AgRP1 suppression of MC4R activity is essential for larval growth (Zhang et al., 2012). Therefore, melanocortin system seems to impose a constitutive break to feeding and growth in fish probably through constitutive activity of MC4R. AgRP1 overexpression is the soundest response to fasting reported in fish, and its hypothalamic expression is essential to counteract MC4R negative effects on energy balance by inverse agonism on the receptor thus driving fish to feeding and concomitantly enhancing fish growth both dependently and independently on feeding levels (Guillot et al., 2016). In fact, AgRP1 mRNA abundance in hypothalamus increased in food deprived zebrafish (Song et al., 2003), goldfish (Cerdá-Reverter and Peter, 2003), sea bass (Agulleiro et al., 2013), and carp (Zhong et al., 2013) but not in Atlantic salmon (Murashita et al., 2009). Post-feeding did not induce changes in AgRP1 hypothalamic mRNA abundance in medaka (Chisada et al., 2014), but the increase in food intake in the GH-transgenic carp is associated with increased values of AgRP1 mRNA (Zhong et al., 2013).

There are no studies on NPY/AgRP colocalization in hypothalamic neurons in fish brain. The NPY family of peptides consists of 36-amino-acid peptides exhibiting carboxy terminal (C-terminal) amidation (Cerdá-Reverter and Larhammar, 2000). The family comprises three different peptides, the NPY, tyrosine-tyrosine peptide, (PYY), and the pancreatic polypeptide (PP). Tetrapod species produce all three peptides, whereas non-tetrapod vertebrates have only NPY and PYY. Teleost fish synthesize two different versions of NPY and PYY but not PP (Sundström et al., 2008). Brain distribution of NPY peptides was reported in detail only in the sea bass by *in situ* hybridization (Cerdá-Reverter et al., 2000a,b), and only NPY1 expressed in the rostral hypothalamus, where the AgRP1 neurons are localized in this species. Concomitant coexpression in the rostral hypothalamus suggests that both NPY1 and AgRP1 colocalize also in the tuberal hypothalamus, ventral telencephalon and preoptic area of fish.

NPY is the most potent orexigenic factor in vertebrates (Stanley and Leibowitz, 1985) and many studies shown this effect after ICV administration in several fish (López-Patiño et al., 1999; Aldegunde and Mancebo, 2006; Kiris et al., 2007; Yokobori et al., 2012). The mRNA abundance of NPY decreased after meal in goldfish (Kehoe and Volkoff, 2007), zebrafish (Tian et al., 2015), and grass carp (Zhou et al., 2013), though responses were contradictory in other species, such as orange-spotted grouper (Tang et al., 2013), rainbow trout (Gong and Björnsson, 2014), and zebrafish (Chen et al., 2016). Food deprivation in rainbow trout also resulted in decreased mRNA abundance of NPY (Gong et al., 2016b) but also after NPY intraperitoneal (IP) injections in flounder (Li et al., 2016). Accordingly, brain expression is upregulated by fasting in several fish species (Matsuda et al., 2012b) but the number of NPY immunoreactive neurons increase in the hypothalamus and posterior tuberculum after seven days fasting in the goldfish but not in the thalamus (Yokobori et al., 2012). Peripheral leptin is also able to regulate hypothalamic NPY expression since IP injection of recombinant leptin inhibits AgRP1/NPY but upregulates POMC/CART expression in goldfish (Yan et al., 2016). In addition, NPY was suggested to link the reproductive system and the central circuitry regulating energy balance in sea bass (Cerdá-Reverter et al., 1999).

## Hypothalamic integration of metabolic information

The detection of nutrient levels in hypothalamus modulates different neurocircuits related to food intake regulation, metabolite homeostasis, energy expenditure, and status of body reserves (Morton et al., 2006; Blouet and Schwartz, 2010). The neurons coexpressing NPY/AgRP or POMC/CART are included in these circuits, and respond with decreased or increased peptide expression, respectively, to rises in circulating levels of glucose, fatty acids, or amino acids (Mobbs et al., 2005; Blouet and Schwartz, 2010; Efeyan et al., 2015). Thus, POMC/CART neurons depolarize while NPY/AgRP hyperpolarize in response to the increase in nutrient levels (Levin et al., 2004; Fioramonti et al., 2007).

The sensing of a particular nutrient may involve the direct binding of the sensed molecule to the sensor, or occur by an indirect mechanism relying on the detection of a related molecule that reflects nutrient abundance (Efeyan et al., 2015). Different organisms detect extracellular and intracellular levels of sugars, amino acids, and fatty acids. We provide a picture of the current knowledge of the systems already characterized in fish, i.e., those involved in the sensing of glucose and fatty acids (Soengas, 2014).

As for amino acids, in mammals the increase in the levels of branched-chain amino acids (BCAA) such as leucine result in decreased food intake. This effect is mediated by activation of central amino acid sensing through changes in target of rapamycin (mTOR) and/or AMP-activated protein-kinase (AMPK), and BCAA metabolism (Heeley and Blouet, 2016; Morrison et al., 2016). Furthermore, the deficiency in essential amino acids elicit an increase in food intake through central amino acids sensors mediated by general control non-depressable 2 (GCN2) and eukaryotic initiation factor 2α (eiF2α) (Fromentin et al., 2012; Maurin et al., 2014). Considering that most fish are carnivorous (strongly dependent on dietary protein/amino acid), the presence of amino acid sensors in central areas involved in the regulation of food intake like hypothalamus is likely. However, no studies are available in fish about the presence and functioning in central areas of amino acid sensors. The only available information relates to the effect of different protein levels and/or composition on food intake with contradictory responses observed (Figueiredo-Silva et al., 2012b; Wacyk et al., 2012; Tan et al., 2016).

**Figure 2:**
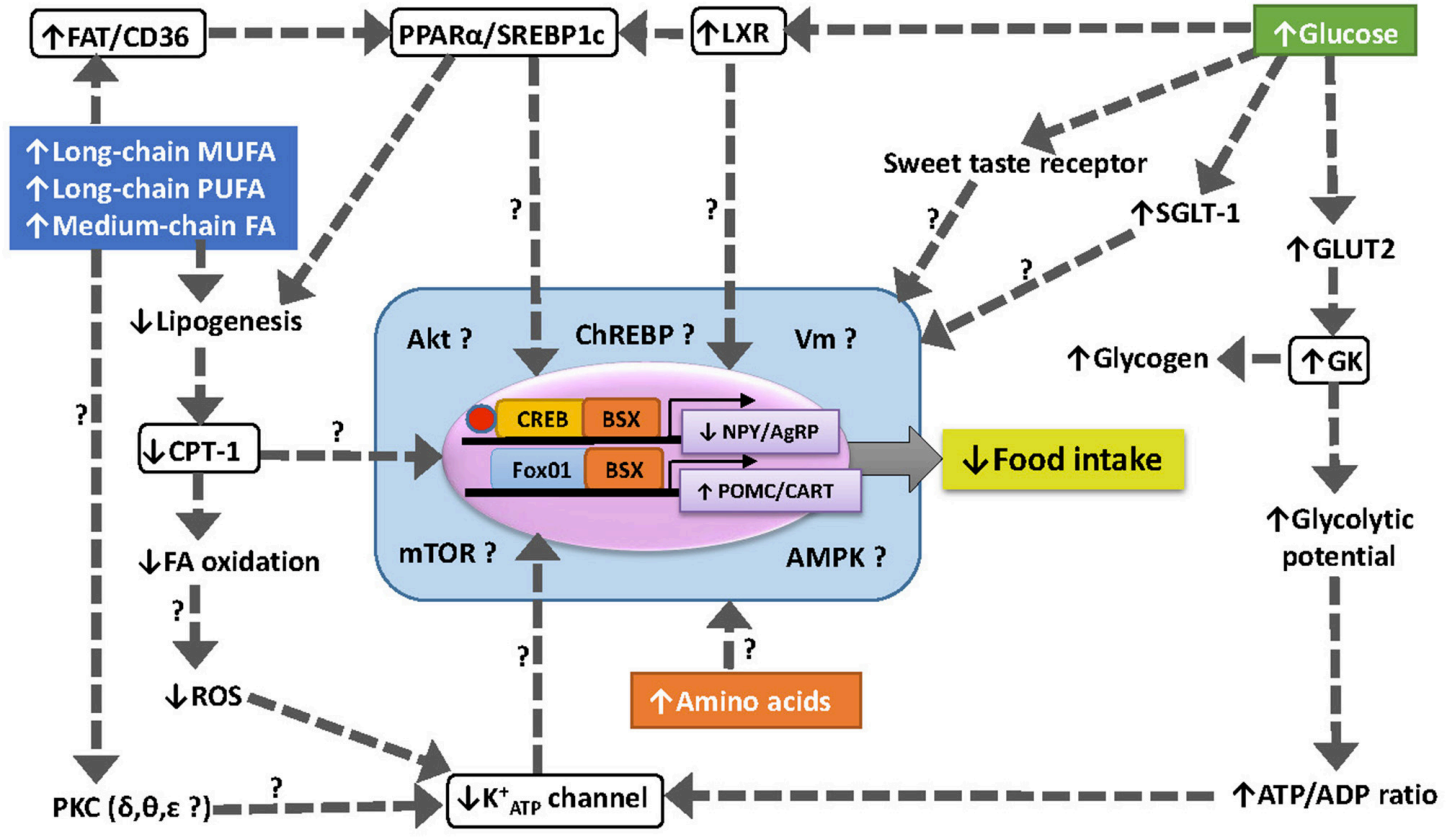
Schematic drawing with a hypothetical model of food intake regulation through different nutrient sensing mechanisms in fish hypothalamus. The increase in levels of specific fatty acids is sensed through several mechanisms including (1) inhibition of CPT-1 to import FA-CoA into the mitochondria for oxidation, (2) increased binding to FAT/CD36 resulting in the modulation of PPARα and SREBP1c, (3) the translocation and activation of specific isoforms of PKC, and (4) the enhanced ROS production in mitochondria by electron leakage. The increase in levels of glucose is sensed through increased GK activity, activation of LXR and modulation o sweet taste receptors. The integration of different sensing mechanisms result in changes in the expression of neuropeptides controlling food intake (NPY/AgRP and CART/POMC) through unknown mechanisms.↑, increase; ↓, decrease; ?, unknown; AgRP, agouti-related peptide; Akt, protein kinase B; AMPK, AMP-activated protein kinase; BSX, brain homeobox transcription factor; CART, cocaine- and amphetamine-related transcript; ChREBP, carbohydrate-responsive element-binding protein; CPT-1, carnitine palmitoyl transferase type 1; CREB, cAMP response-element binding protein FA, fatty acid; FAT/CD36, fatty acid translocase; FoxO1, forkhead box protein O1; K^+^_ATP_, inward rectifier ATP-dependent K^+^ channel; GK, glucokinase (hexokinase IV); GLUT2, facilitative glucose carrier type 2; LXR, liver X receptor; mTOR, target of rapamycin; MUFA, monounsaturated fatty acid; NPY, neuropeptide Y; POMC, pro-opio melanocortin; PPARα, peroxisome proliferator-activated receptor type α; PUFA, polyunsaturated fatty acid; SGLT-1, sodium/glucose co-transporter 1; SREBP1c, sterol regulatory element-binding protein type 1c; PKC, protein kinase C; ROS, reactive oxygen species; Vm, membrane potential.

A summary of the main findings achieved as well as the hypothetical pathways involved in nutrient sensing in fish hypothalamus is shown in Figure 2.

### Glucosensors and control of food intake

#### Glucosensing mechanisms

Hypothalamic POMC/CART neurons increase and NPY/AgRP neurons decrease their firing rate in response to increased levels of glucose in mammals (Levin et al., 2004; Marty et al., 2007). This process is glucosensing. The best characterized glucosensing mechanism is that dependent on glucokinase (GK), and is similar to that existing in β-cells of endocrine pancreas (Blouet and Schwartz, 2010; Efeyan et al., 2015). Glucose is transported into the cell by glucose facilitative carrier type 2 (GLUT2) and then phosphorylated by GK. Once phosphorylated, glucose is metabolized through glycolysis then increasing intracellular ATP/ADP ratio leading to the closure of the ATP-dependent inward rectified potassium channel (K^+^_ATP_), membrane depolarization, calcium entry through L-type voltage-dependent calcium channel, and increased neuronal activity (Marty et al., 2007; De Backer et al., 2016). However, since not all glucosensing neurons rely on this mechanism (Fioramonti et al., 2004; De Backer et al., 2016), evidence for the existence of alternative glucosensing mechanisms obtained in several studies. Thus, high glucose concentrations stimulate the expression of liver X receptor (LXR) (Mitro et al., 2007) resulting in an inhibition of gluconeogenesis (Anthonisen et al., 2010; Archer et al., 2014). The stimulation by glucose of sweet taste receptors (similar to those in lingual taste cells) depending on a heterodimer of type 1 taste receptor subunits (T1Rs) formed by T1R2 + T1R3 and the G protein α-gustducin activates an intracellular signaling cascade (Ren et al., 2009). The expression of sodium/glucose co-transporter 1 (SGLT-1) increases in response to enhanced glucose levels (Díez-Sampedro et al., 2003; González et al., 2009; Herrera Moro Chao et al., 2016). Finally, another mechanism relies on mitochondrial production of reactive oxygen species leading to increased expression of uncoupling protein 2 in response to increased glucose levels (Blouet and Schwartz, 2010). In addition, several of these systems appear to be inter-connected. Thus, for instance, T1R3 and α-gustducin are necessary for increased SGLT-1 induction by dietary carbohydrates (Wauson et al., 2013).

In fish, recent studies demonstrated the presence and functioning of components of a GK-dependent glucosensing mechanism in brain areas of different species (Polakof et al., 2011d, 2012; Soengas, 2014). The response in rainbow trout hypothalamus after IP, ICV or dietary treatments inducing changes in the levels of glucose is shown in Table 1. Moreover, recent studies (Otero-Rodiño et al., 2015, 2016) provided evidence in rainbow trout for the presence and response to changes in circulating levels of glucose of glucosensing mechanisms in hypothalamus dependent on mitochondrial activity, LXR, and sweet taste receptor. No other studies attempted to elucidate the presence of glucosensing mechanisms in fish though glucose-sensing properties have been recently described in medaka hypothalamus (Hasebe et al., 2016).

**Table 1:** Effects in different fish species of different treatments eliciting changes in glucose levels (change) on food intake (FI), the response of hypothalamic glucosensing systems (sensing), and the mRNA abundance in hypothalamus of orexigenic (NPY, AgRP) and anorexigenic (POMC, CART) neuropeptides.

**Treatment**	**Change**	**Dose**	**Time**	**Species**	**FI**	**Sensing**	**NPY**	**AgRP**	**POMC**	**CART**	**References**
**GLUCOSE**											
Oral	↑	1 g/mL	4 h	Rainbow trout	↓						Soengas and Aldegunde, 2004
IP	↑	500 mg/kg	3 h	Rainbow trout	▾						Ruibal et al., 2002
	↑	500 mg/kg	6 h	Rainbow trout		▴					Polakof et al., 2007a
	↑	500 mg/kg	6 h	Rainbow trout		▴	▾		▴	▴	Conde-Sieira et al., 2010b
	↑	500 mg/kg	6 h	Rainbow trout		▴					Conde-Sieira et al., 2012b
	↑	500 mg/kg	6 h	Rainbow trout		▴	≈	≈	↑	↑	Otero-Rodiño et al., 2015
	↑	500 mg/kg	2–5 days	Rainbow trout	↓	▴					Conde-Sieira et al., 2010a
	↑	500 mg/kg	1–10 days	Rainbow trout	↓	▴					Polakof et al., 2008a
ICV		100 ng/g	2 h	Catfish						▴	Subhedar et al., 2011
		4 μg/g	6 h	Rainbow trout		↑					Polakof and Soengas, 2008
*In vitro*	↑	2–8 mM	1 h	Rainbow trout		▴					Polakof et al., 2007b
	↑	2–8 mM	1 h	Rainbow trout		▴	▾		▴	▴	Aguilar et al., 2011
	↑	2–8 mM	1 h	Rainbow trout		↑	↓		≈	↑	Conde-Sieira et al., 2011
	↑	2–8 mM	1 h	Rainbow trout		↑	↓		≈	≈	Conde-Sieira et al., 2012a
	↑	2–8 mM	6 h	Rainbow trout		↑					Otero-Rodiño et al., 2016
Carbohydrate-enriched diet	↑	35–55% CHO	0.5 h	Goldfish	↓		↓				Narnaware and Peter, 2002
	↑	31% CHO	1–6 h	Rainbow trout			≈			↑	Figueiredo-Silva et al., 2012b
	↑	20% CHO	1–24 h	Rainbow trout		▴					Polakof et al., 2008c
	↑	20% CHO	1–10 days	Rainbow trout	↓	▴					Polakof et al., 2008b
	↑	20% CHO	1–10 days	Rainbow trout		▴					Polakof et al., 2008c
	↑	23% CHO	28 days	Rainbow trout	↓						Krogdahl et al., 2004
	↑	31% CHO	42 days	Rainbow trout	↓						Figueiredo-Silva et al., 2012b
	↑	31% CHO	42 days	Rainbow trout	↓						Figueiredo-Silva et al., 2013
	↑	27–46% CHO	48 days	Tilapia	↓						Saravanan et al., 2012
	↑	26% CHO	48 days	Tilapia	↓						Figueiredo-Silva et al., 2013
	≈	33% CHO	70 days	Gilthead sea bream	≈						Rocha et al., 2016
	↑	19% CHO	73 days	Sea bass	≈						Castro et al., 2015
	↑	30–40% CHO	84 days	Rainbow trout	↓						Suárez et al., 2002
	↑	38% CHO	126 days	Rainbow trout	↓						Kaushik et al., 1989
Carbohydrate-free diet	↓	<0.3% CHO	1–10 days	Rainbow trout	↑	↓					Polakof et al., 2008b
	↓	<0.3% CHO	1–10 days	Rainbow trout		▾					Polakof et al., 2008c
	↓	<0.1% CHO	28 days	Rainbow trout	↑						Sánchez-Muros et al., 1998
	↓	0.2% CHO	70 days	Rainbow trout	↑						Capilla et al., 2003

*↑, Increase; ▴, strong increase; ↓, decrease; ▾, strong decrease; ≈, no changes; IP, intraperitoneal; ICV, intracerebroventricular; CHO, carbohydrate*.

#### Glucose and food intake control

The detection of changes in glucose levels induces several regulatory responses allowing the animal to control glycemia, and one of these responses is food intake regulation (Marty et al., 2007). Thus, hypo- and hyperglycemia are known to increase and decrease food intake in mammals (Baird et al., 1997; Sanders et al., 2006) and birds (Seino and Miki, 2003), respectively.

Several studies carried out in fish suggest that changes in glucose levels may also modulate food intake response (Polakof et al., 2011d; Soengas, 2014), as summarized in Table 1. In general, fish fed with high-carbohydrate diets, or with glucose levels raised through IP or ICV treatments, displayed a decreased food intake, as demonstrated studies carried out in rainbow trout, goldfish, and tilapia. However, in some studies no changes occurred in food intake, as in sea bass or gilthead sea bream. Furthermore, fish fed a diet without carbohydrates or with low glucose levels resultant of IP or ICV treatments, increased food intake (Polakof et al., 2008a,b).

The mRNA of NPY, AgRP, POMC, and CART was detected in brain of different fish species in areas analogous to those of mammals (Cerdá-Reverter and Canosa, 2009), and changes in mRNA abundance related to food intake control (Volkoff et al., 2009). The presence in the same areas of rainbow trout brain of a marker of glucosesning such as GK protein (Polakof et al., 2009) and mRNA of neuropeptides involved in food intake control suggest that both findings are related. There are however few studies in fish assessing changes in hypothalamic neuropeptide mRNA abundance under conditions of altered glucose levels (Table 1). In rainbow trout NPY mRNA abundance decreased in hypothalamus after glucose treatment (Conde-Sieira et al., 2010b, 2012b; Aguilar et al., 2011; Otero-Rodiño et al., 2015) or after fish were fed with a high carbohydrate diet (Narnaware and Peter, 2002; Figueiredo-Silva et al., 2012b). Moreover, increased CART mRNA levels were observed in hypothalamus after experimental rising of glucose levels in catfish (Subhedar et al., 2011) and rainbow trout (Conde-Sieira et al., 2010b, 2012b; Otero-Rodiño et al., 2015), or in rainbow trout fed with a diet enriched with carbohydrates (Figueiredo-Silva et al., 2012b). Hypothalamic POMC-A1 mRNA abundance also increased in rainbow trout after hyperglycaemic treatment (Conde-Sieira et al., 2010b; Otero-Rodiño et al., 2015). Thus, increased glucose levels elicit increased anorectic potential whereas decreased glucose levels elicit increased orexigenic potential in agreement with the changes in food intake reported in the same species (Polakof et al., 2008a,b).

### Fatty acid sensors and control of food intake

#### Fatty acid sensing mechanisms

Evidence in mammals supports that specialized neurons in hypothalamus detect changes in circulating levels of long-chain fatty acid (LCFA), but not short- (SCFA) or medium-chain fatty acid (MCFA) contributing to neural control of energy homeostasis (Migrenne et al., 2007; Gao et al., 2013; Duca and Yue, 2014; Efeyan et al., 2015). The most accepted mechanism is of metabolic nature. Thus, increased LCFA levels in plasma induced an increase in malonyl-CoA levels and subsequent inhibition of carnitinepalmitoyltransferase 1 (CPT-1) to import FA-CoA into the mitochondria for oxidation (López et al., 2005, 2007). Other fatty acid sensing mechanisms are present in hypothalamus. Thus, the increased binding to fatty acid translocase (FAT/CD36) induced by increased levels of LCFA results in the modulation of transcription factors like sterol regulatory element-binding protein type 1c (SREBP1c), and peroxisome proliferator-activated receptor type α (PPARα) (Le Foll et al., 2009). The translocation and activation of specific isoforms of protein kinase C (PKC) in response to enhanced LCFA levels results in PI3K inhibition (Benoit et al., 2009; Blouet and Schwartz, 2010). The enhanced production of reactive oxygen species in mitochondria in response to raised levels of LCFA results in K^+^_ATP_ inhibition (Blouet and Schwartz, 2010). Finally, the activity of lipoprotein lipase related to increased levels of LCFA (Picard et al., 2013). The 18 carbon monounsaturated fatty acid oleate (C18:1 n-9) is the most studied LCFA in mammals involved in the activation of fatty acid sensing systems (López et al., 2007; Blouet and Schwartz, 2010; Duca and Yue, 2014). Fatty acid unsaturation appears to be important since the saturated fatty acid palmitate (C16:0) does not activate hypothalamic fatty acid sensing systems (Ross et al., 2010; Schwinkendorf et al., 2011; Greco et al., 2014). Moreover, the presence of more than one double bond, such as for linoleate (C18:2 n-6) or docosahexanoate (C22:6 n-3), does not activate fatty acid sensing systems in mammals (Gomez-Pinilla and Ying, 2010; Ross et al., 2010; Schwinkendorf et al., 2011; Greco et al., 2014).

In fish, lipids are main nutrients involved in metabolically sustaining relevant physiological processes (Tocher, 2003; Polakof et al., 2010). Thus, it is reasonable that lipids are involved in food intake control. In recent years, the presence and function of fatty acid sensing systems in the hypothalamus was characterized in fish (Librán-Pérez et al., 2012, 2013, 2014a,b, 2015a,b), as summarized in Table 2. IP (Librán-Pérez et al., 2012), ICV (Librán-Pérez et al., 2014a), and *in vitro* (Librán-Pérez et al., 2013) administration in rainbow trout of oleate or the MCFA octanoate (C8:0) induced a response in hypothalamus compatible with fatty acid sensing. This included reduced potential of lipogenesis and fatty acid oxidation, decreased potential of K^+^_ATP_, and modulation of FAT/CD36 with subsequent changes in the expression of transcription factors (Librán-Pérez et al., 2012, 2013, 2014a). This response is comparable with that of mammals with the main difference of the capacity of fish to respond to increased levels of an MCFA like octanoate (Hu et al., 2011). This could relate to the finding that body lipids in teleosts contain considerable amounts of MCFA (Davis et al., 1999; Trushenski, 2009) and that in rainbow trout there is no preferential oxidation of MCFA compared with LCFA (Figueiredo-Silva et al., 2012a), in contrast with the mammalian situation (Ooyama et al., 2009).

**Table 2:** Effects in different fish species of different treatments eliciting changes in fatty acid levels (change) on food intake (FI), the response in hypothalamus of fatty acid sensing systems (sensing), and the mRNA abundance in hypothalamus of orexigenic (NPY, AgRP) and anorexigenic (POMC, CART) neuropeptides.

**Treatment**	**Change**	**Dose**	**Time**	**Species**	**FI**	**Sensing**	**NPY**	**AgRP**	**POMC**	**CART**	**References**
**OLEATE**											
IP	↑	300 μg/kg	3 h	Senegalese sole		▴	≈	▾	≈	▴	Conde-Sieira et al., 2015
	↑	60–300 μg/kg	6 h	Rainbow trout		▴	▾		▴	↓	Librán-Pérez et al., 2012
	↑	300 μg/kg	6 h	Rainbow trout		↑	≈		≈	≈	Librán-Pérez et al., 2015a
	↑	60–300 μg/kg	6–24 h	Rainbow trout	▾						Librán-Pérez et al., 2012
	↑	300 μg/kg	6–24 h	Rainbow trout	↓						Librán-Pérez et al., 2015a
	↑	300 μg/kg	4–48 h	Senegalese sole	↓						Conde-Sieira et al., 2015
ICV	↑	10 nmol/g	2–6 h	Rainbow trout		↑	↓	↓	▴	↑	Velasco et al., 2016a
	↑	10 nmol/g	6 h	Rainbow trout		↑	▾		▴	↑	Librán-Pérez et al., 2014a
	↑	10 nmol/g	6–24 h	Rainbow trout	▾						Librán-Pérez et al., 2014a
	↑	10 nmol/g	6–48 h	Rainbow trout	▾						Velasco et al., 2016a
*In vitro*	↑	1–100 μM	1 h	Rainbow trout		▴	▾		▴	▴	Librán-Pérez et al., 2013
Stearate (IP)	↑	300 μg/kg	3 h	Senegalese sole		≈	≈	≈	≈	↓	Conde-Sieira et al., 2015
	↑	300 μg/kg	4–48 h	Senegalese sole	≈						Conde-Sieira et al., 2015
α-Linolenate (IP)	↑	300 μg/kg	3 h	Senegalese sole		▴	≈	▾	≈	↑	Conde-Sieira et al., 2015
	↑	300 μg/kg	4–48 h	Senegalese sole	↓						Conde-Sieira et al., 2015
Eicosapentanoate (IP)	↑	300 μg/kg	3 h	Senegalese sole		≈	≈	≈	≈	▴	Conde-Sieira et al., 2015
	↑	300 μg/kg	4–48 h	Senegalese sole	↓						Conde-Sieira et al., 2015
**OCTANOATE**											
IP	↑	60–300 μg/kg	6 h	Rainbow trout		▴	≈		≈	↓	Librán-Pérez et al., 2012
	↑	300 μg/kg	6 h	Rainbow trout		↑	≈		≈	≈	Librán-Pérez et al., 2015a
	↑	60–300 μg/kg	6–24 h	Rainbow trout	▾						Librán-Pérez et al., 2012
	↑	60–300 μg/kg	6–24 h	Rainbow trout	↓						Librán-Pérez et al., 2015a
ICV	↑	10 nmol/g	2–6 h	Rainbow trout		↑	↓		▴	≈	Librán-Pérez et al., 2014a
	↑	10 nmol/g	6–24 h	Rainbow trout	▾						Librán-Pérez et al., 2014a
*In vitro*	↑	1–100 μM	1 h	Rainbow trout		▴	≈		▴	▴	Librán-Pérez et al., 2013
Lipid- enriched diet	↑	20% Lipid	1–6 h	Rainbow trout				↓		≈	Figueiredo-Silva et al., 2012b
	↑	25–34% Lipid	1–10 days	Rainbow trout	≈						Geurden et al., 2006
	↑	21% Lipid	21 days	Rainbow trout	≈						Luo et al., 2014
	↑	19% Lipid	28 days	Rainbow trout	≈	↑	↑	≈	▴	▴	Librán-Pérez et al., 2015b
	↑	30–40% Lipid	40 days	Rainbow trout	↓						Forsman and Ruohonen, 2009
	↑	19–27% Lipid	42 days	Rainbow trout	↓						Saravanan et al., 2013
	↑	20% Lipid	42 days	Rainbow trout	↓						Figueiredo-Silva et al., 2012b
	↑	20% Lipid	42 days	Rainbow trout	↑						Figueiredo-Silva et al., 2013
	↑	21% Lipid	48 days	Tilapia	≈						Figueiredo-Silva et al., 2013
	↑	18% Lipid	50 days	Rainbow trout	↓						Peragón et al., 2000
	↑	35% Lipid	56 days	Polka-dot grouper	↓						Williams et al., 2006
	↑	34% Lipid	56 days	Atlantic salmon	↓		≈	≈	≈	↑	Hevroy et al., 2012
	↑	21% Lipid	56 days	Grass carp	↓						Li et al., 2016
	↑	30% Lipid	63–84 days	Rainbow trout	↓						Gélineau et al., 2001
	↑	21% Lipid	105 days	Rainbow trout	≈						Figueiredo-Silva et al., 2012a
SDW WAG994 (IP)	↓	60 mg/kg	6 h	Rainbow trout		↓	≈		▾	▾	Librán-Pérez et al., 2014b
	↓	60 mg/kg	24–48 h	Rainbow trout	↑						Librán-Pérez et al., 2014b
Insulin (IP; het.)	↓	2 mg/kg	6 h	Rainbow trout		↑	≈		≈	↓	Librán-Pérez et al., 2015a
	↓	2 mg/kg	6–24 h	Rainbow trout	▾						Librán-Pérez et al., 2015a
Ghrelin (ICV; hom.)	↑	2 ng/g	2–6 h	Rainbow trout		↓	↑	↑	↓	↓	Velasco et al., 2016a
	↑	2 ng/g	6–48 h	Rainbow trout	↑						Velasco et al., 2016a

*↑, Increase; ▴, strong increase; ↓, decrease; ▾, strong decrease; ≈, no changes; IP, intraperitoneal; ICV, intracerebroventricular; hom., homologous hormone; het. Heterologous hormone*.

All vertebrate species have dietary requirements for specific polyunsaturated fatty acid (PUFA), and diets for marine fish are particularly rich in long chain PUFA (Sargent et al., 2002). The brain of marine fish displays high levels of n-3 PUFA, mainly in α-linolenate (C18:3 n-3), eicosapentanoate (C20:5 n-3), and docosahexanoate (Tocher et al., 1992; Betancor et al., 2014). Therefore, it is reasonable to hypothesize that fish hypothalamic FA sensing systems, particularly in marine species, could differ from those of mammals in the ability to sense PUFA. Accordingly, a recent study in Senegalese sole (Conde-Sieira et al., 2015) demonstrated that its fatty acid sensing systems were activated not only by oleate but also by an n-3 PUFA such as α-linolenate. However, in the same study authors showed that another PUFA such as eicosapentanoate did not alter fatty acid sensing systems suggesting that the response might be specific to certain PUFA.

If fatty acid sensing systems activated when levels of specific fatty acid rise, what happens in those systems when levels of fatty acids fall? It is not possible to decrease the levels of a particular fatty acid. The only available studies in mammals used pharmacological treatments inhibiting lipolysis to induce a general decline in levels of all circulating fatty acids, which coincided with decreased activity of fatty acid sensing systems (Oh et al., 2012, 2014). A similar experimental approach in rainbow trout resulted in an inhibition of fatty acid sensing mechanisms in hypothalamus associated with the activation of the hypothalamus-pituitary-interrenal (HPI) axis (Librán-Pérez et al., 2014b).

#### Fatty acids and control of food intake

Feeding fish with diets enriched in lipids result in a decrease in food intake as observed in different species (Table 2). A comparable lower food intake also occurred in fish containing high fat stores (Shearer et al., 1997; Silverstein et al., 1999; Johansen et al., 2002, 2003). Considering the qualitative and quantitative importance of fatty acids within the lipid pool in fish diets and tissue composition, it is not surprising that the available studies are focussed on fatty acids.

In recent studies in rainbow trout, increased levels of oleate or octanoate *in vivo*, either after IP (Librán-Pérez et al., 2012) or ICV (Librán-Pérez et al., 2014a) treatments, resulted in decreased food intake, with more potent effects for octanoate. Moreover, also in rainbow trout fed diets with different lipid composition, the fish with the highest levels of fatty acid in plasma were those in which a decreased food intake occurred (Luo et al., 2014). Therefore, the inhibition of food intake reported in fish by feeding lipids is probably due to the action of central fatty acid sensors. This is also supported by the finding that the treatment of rainbow trout with C75 (fatty acid synthase inhibitor) resulted in a reduced food intake counteracted by TOFA (acetyl-CoA carboxylase inhibitor) (Librán-Pérez et al., 2012). In Senegalese sole, a decreased food intake occurred after IP treatment with oleate as well as different types of fatty acids including saturated fatty acids like stearate, and two types of PUFA such as α-linolenate and eicosapentanoate (Conde-Sieira et al., 2015). When levels of circulating fatty acids decreased through non-specific pharmacological treatment, a sound increase in food intake occurred in rainbow trout (Librán-Pérez et al., 2014b).

In mammals, enhanced levels of LCFA resulted in a decrease in mRNA abundance of AgRP and NPY as well as an increase of CART and POMC (López et al., 2005). However, few studies assessed in fish hypothalamus mRNA abundance of neuropeptides in response to changes in levels of fatty acids in circulation (Table 2). In rainbow trout the treatment with oleate or octanoate either IP (Librán-Pérez et al., 2012), ICV (Librán-Pérez et al., 2014a), or *in vitro* (Librán-Pérez et al., 2013) resulted in a decrease in mRNA abundance of NPY and an increase of CART and POMC-A1. The decrease in NPY is comparable to that described in rat hypothalamus after oleate ICV treatment (Blouet and Schwartz, 2010). Changes in mRNA abundance of neuropeptides suggest an enhancement of anorexigenic potential, which is in agreement with the decrease observed in food intake of rainbow trout after oleate or octanoate treatment (Librán-Pérez et al., 2012, 2014a). It is important to emphasize that the response to octanoate is unique to fish since in mammals octanoate is not inducing any change in mRNA abundance of neuropeptides (Hu et al., 2011). In Senegalese sole, the increase in circulating levels of oleate also resulted in decreased mRNA abundance of AgRP-2 and increased values of CART-2b again favoring increased anorectic potential (Conde-Sieira et al., 2015). In the same species the IP treatment with PUFA such as α-linolenate or eicosapentanoate (Conde-Sieira et al., 2015) increased anorectic potential based on decreased mRNA abundance of AgRP-2 (α-linolenate) and increased mRNA abundance of CART-2b (α-linolenate and eicosapentanoate). The decrease in circulating levels of fatty acid induced in rainbow trout by pharmacological treatment resulted in a fall of anorexigenic potential based on decreased mRNA abundance of POMC-A1 and CART (Librán-Pérez et al., 2014b).

### Integration of nutrient sensing information

The precise mechanisms connecting changes in glucose or fatty acid sensing systems and the mRNA abundance of orexigenic and anorexigenic factors are mostly unknown. Changes in neuropeptide expression have been associated with the modulation of brain homeobox transcription factor (BSX), forkhead box01 (Fox01), and phosphorylated cAMP response-element binding protein (pCREB) (Diéguez et al., 2011). The actions of these factors would result in the enhancement of CART and POMC, and the inhibition of NPY and AgRP, ultimately leading to decreased food intake (López et al., 2007; Diéguez et al., 2011). How these transcription factors relate to the activity of the different nutrient sensing systems? Several possibilities were suggested in mammals including (1) direct action of malonyl CoA or CPT1c, (2) indirect action through inhibition of CPT-1, (3) modulation by protein kinase B (Akt), AMPK, carbohydrate-responsive element-binding protein (ChREBP), or mTOR, or (4) involvement of ceramides (López et al., 2007; Diéguez et al., 2011; Gao et al., 2013).

In fish, a preliminary study in rainbow trout (Librán-Pérez et al., 2015b) showed that the cell signaling pathways that are dependent on Akt, AMPK, and mTOR activated in hypothalamus of fish fed a lipid-enriched diet. In the same species, a recent study (Velasco et al., 2016b) pointed to a possible role of ceramides in connecting hypothalamic fatty acid sensing systems and neuropeptide mRNA abundance with food intake control. Besides these studies, there is no further evidence in fish about the integration of the metabolic information of different nutrient (glucose, fatty acids, amino acids) sensing systems into a unique pathway regulating transcription factors involved in the production of neuropeptides controlling food intake. A summary of hypothetical relationships is shown in Figure 2.

## Hypothalamic integration of endocrine information

The hypothalamic neurons producing neuropeptides that control food intake in response to changes in the levels of nutrients are also responsive, through binding to appropriate receptors, to the effect of hormones (Levin et al., 2004; Blouet and Schwartz, 2010). These include hormones providing information regarding metabolic stores or energy *status* such as leptin and insulin, and gastrointestinal hormones providing information regarding absence/presence of food and its composition, including ghrelin and cholecystokinin (CCK), among others (Blouet and Schwartz, 2010). The same hormones modulate the activity of fatty acid- and glucose-sensing systems and mRNA abundance of neuropeptides related to the control of food intake in hypothalamus, as summarized in Table 2 (fatty acid) and Table 3 (glucose).

**Table 3:** Effects in different fish species of different hormone (either homologous: hom., or heterologous: het.) treatments eliciting changes in glucose levels (change) on food intake (FI), the response of hypothalamic glucosensing systems (sensing), and the mRNA abundance in hypothalamus of orexigenic (NPY, AgRP) and anorexigenic (POMC, CART) neuropeptides.

**Treatment**	**Change**	**Dose**	**Time**	**Species**	**FI**	**Sensing**	**NPY**	**AgRP**	**POMC**	**CART**	**References**
**INSULIN**											
IP	↓	4 mg/kg (het.)	6 h	Rainbow trout		▾					Polakof et al., 2007a
	↓	4 mg/kg (het.)	6 h	Rainbow trout		↓	≈		≈	≈	Conde-Sieira et al., 2010b
	↓	4 mg/kg (het.)	2–5 days	Rainbow trout	↑	↓					Conde-Sieira et al., 2010a
	↓	4 mg/kg (het.)	1–10 days	Rainbow trout	↑	↓					Polakof et al., 2008a
ICV		104 ng/fish (het.)	2 h	Catfish						↑	Subhedar et al., 2011
		20 ng/g (het.)	1–24 h	Catfish	≈						Silverstein and Plisetskaya, 2000
		90 ng/g (het.)	26–50 h	Rainbow trout	↓						Soengas and Aldegunde, 2004
**GLP-1**											
IP	≈	50–200 ng/g (het.)	1 h	Channel catfish	≈		≈		≈		Schroeter et al., 2015
	↑	100 μg/kg (het.)	2 h	Rainbow trout		▴	↑		▴	≈	Polakof et al., 2011b
		50–300 ng/g (het.)	4 h	Coho salmon	▾						White et al., 2016
ICV	↑	20 ng/g (het.)	1 h	Rainbow trout		≈	≈		↓	≈	Polakof et al., 2011b
		5–50 ng/g (hom.)	1 h	Catfish	▾						Silverstein et al., 2001
*In vitro*		0.1–10 nM (het.)	1 h	Rainbow trout		↑	↓		↑	↑	Polakof et al., 2011b
**LEPTIN**											
IP		120 ng/g (hom.)	1–8 h	Rainbow trout	▾		▾		▴	↑	Murashita et al., 2008
	↓	0.1–3.3 μg/g (het.)	2–8 h	Goldfish	▾						De Pedro et al., 2006
	↑	1 μg/g (het.)	8 h	Goldfish	▾						Vivas et al., 2011
ICV		50 ng/g (het.)	1 h	Goldfish	▾						Volkoff and Peter, 2001a
		1–100 ng/g (het.)	1 h	Goldfish	↓						Volkoff et al., 2003
		40 ng/g (het.)	1 h	Catfish	≈						Silverstein and Plisetskaya, 2000
		10 ng/g (het.)	2 h	Catfish						↑	Subhedar et al., 2011
		0.5–5 ng/g (hom.)	2 h	Rainbow trout	▾		↑		▴	↑	Gong et al., 2016a
		50 ng/g (het.)	2–6 h	Goldfish			↓			↑	Volkoff and Peter, 2001a
	↑	50 ng/g (het.)	6 h	Rainbow trout		▴					Aguilar et al., 2010
	↑	50 ng/g (het.)	6–24 h	Rainbow trout	▾						Aguilar et al., 2010
*In vitro*	↑	10–50 nM (het.)	1–3 h	Rainbow trout		▴	▾		≈	≈	Aguilar et al., 2011
**GHRELIN**											
IP		100 ng/g (het.)	0.5 h	Cavefish	▴					≈	Penney and Volkoff, 2014
	≈	200 ng/g (hom.)	1 h	Rainbow trout	↓						Jönsson et al., 2010
	≈	50–200 ng/g (hom.)	1 h	Channel catfish	▾		≈		≈		Schroeter et al., 2015
		100 μg/kg (het.)	2 h	Rainbow trout		▴	≈		≈	≈	Polakof et al., 2011c
		16 pmol/g (hom.)	1–6 h	Goldfish			≈				Miura et al., 2006
		475 ng/g (hom.)	7 days	Brown trout	▴		≈				Tinoco et al., 2014a
ICV		1 pmol/g (hom.)	1 h	Goldfish	▴						Miura et al., 2006
	≈	20 ng/g (het.)	2 h	Rainbow trout		≈	≈		≈	≈	Polakof et al., 2011c
		1 pmol/g (hom.)	1–6 h	Goldfish			↑				Miura et al., 2006
*In vitro*		0.1–10 nM (het.)	1 h	Rainbow trout		↑	↑		≈	↓	Polakof et al., 2011c
**CCK**											
IP		100 ng/g (het.)	0.5 h	Goldfish	▾						Himick and Peter, 1994
		50 ng/g (het.)	0.5 h	Cavefish	▾						Penney and Volkoff, 2014
		50–200 ng/g (het.)	1 h	Channel catfish	≈		≈		↓		Schroeter et al., 2015
	↑	50 μg/kg (het.)	2 h	Rainbow trout		≈					Polakof et al., 2011a
		50–500 ng/g (het.)	4 h	Coho salmon	▾					↓	White et al., 2016
ICV		50 ng/g (het.)	0.5 h	Goldfish	▾						Himick and Peter, 1994
		50–100 pmol/g (het.)	1 h	Goldfish	↓				▴		Kang et al., 2010
		10 ng/g (het.)	1 h	Catfish	▾						Silverstein and Plisetskaya, 2000
	↑	10 ng/g (het.)	2 h	Rainbow trout		↑					Polakof et al., 2011a

*↑, Increase; ▴, strong increase; ↓, decrease; ▾, strong decrease; ≈, no changes; IP, intraperitoneal; ICV, intracerebroventricular*.

### Insulin

Insulin and insulin receptors are present in fish hypothalamus (Gutiérrez and Plisetskaya, 1994; Leibush et al., 1996; Caruso et al., 2008). The effects of insulin treatment on food intake in fish are however contradictory. Thus, in rainbow trout IP administration of insulin either resulted in inhibition (Librán-Pérez et al., 2015a) or activation (Polakof et al., 2008a; Conde-Sieira et al., 2010b) of food intake, whereas ICV treatment did not affect food intake in catfish (Silverstein and Plisetskaya, 2000) but induced a decrease in rainbow trout (Soengas and Aldegunde, 2004). The anorectic effects in rainbow trout agree with the increased anorexigenic potential (increased CART and decreased NPY mRNA abundance) observed in hypothalamus after insulin treatment (Librán-Pérez et al., 2015a). Moreover, insulin administration in rainbow trout inhibits the glucosensing system dependent on GK (Polakof et al., 2007a, 2008a; Conde-Sieira et al., 2010b) whereas no clear effects were noted on fatty acid sensing systems (Librán-Pérez et al., 2015a).

### Leptin

Multiple forms of leptin exist in fishes, with the major form, leptin A, being the most likely candidate for food intake regulation and energy signaling (Gorissen and Flik, 2014). The treatment with either fish or recombinant human leptin in fish usually results in an anorectic response (De Pedro et al., 2006; Murashita et al., 2008; Kling et al., 2009; Vivas et al., 2011; Won et al., 2012; Gong et al., 2016a). This anorexigenic action of leptin appears to be mediated by brain leptin receptors, particularly in the hypothalamus (Kurokawa et al., 2008; Tinoco et al., 2012; Angotzi et al., 2016), where leptin exerts its anorectic action through regulation of neuropeptide expression. Accordingly, the leptin receptor-deficient medaka shows increased food intake accompanied by increased NPYa and AgRP mRNA abundance, and decreased POMC1 mRNA abundance (Chisada et al., 2014). Moreover, ICV injection of leptin reduced NPY mRNA levels in hypothalamus and telencephalon of goldfish (Volkoff et al., 2003), and in the whole brain of grass carp (Li et al., 2010). Peripheral treatment with recombinant salmon leptin A1 in rainbow trout induced a hypothalamic transient reduction and elevation of NPY and POMC-A1 mRNA levels, respectively (Murashita et al., 2011). Moreover, central injection of leptin increased CART-I mRNA levels in hypothalamus of goldfish (Volkoff and Peter, 2001a), and POMC-A1, A2 and B, and CART mRNA levels in rainbow trout (Gong et al., 2016a). On the other hand, the leptin anorectic effects also related in rainbow trout to the activation of central glucosensing systems (Aguilar et al., 2010, 2011).

The action of leptin on food intake regulation depends on the time of hormone administration, indicating a circadian dependence of leptin anorexigenic effects (Vivas et al., 2011). In this sense, leptin-aI and leptin-aII mRNAs in hypothalamus of goldfish show 24-h rhythms under light–dark cycle and scheduled feeding conditions (Tinoco et al., 2014b). Post-prandial increases of hypothalamic and liver leptin-aI expression (Huising et al., 2006; Moen and Finn, 2013; Zhang et al., 2013) are in agreement with the anorexigenic role of this hormone in fish. As expected for an anorexigenic hormone, feeding changes and nutritional status modifies the leptin system in fish, but different responses occurred depending on the feeding regime and species. Thus, leptin genes expression or circulating leptin levels rise with food restriction or starvation, when energy stores decline, and decrease during refeeding in some species (Gorissen and Flik, 2014; Johansson and Björnsson, 2015), but not in others (Huising et al., 2006; Tinoco et al., 2012). These, and other recent studies (Chisada et al., 2014; Londraville et al., 2014; Salmerón et al., 2015; Jørgensen et al., 2016) support that leptin in fish should not be considered as a lipostat signal in contrast to mammals. In this way, zebrafish knockout for leptin suggest the involvement of leptin in glucose homeostasis but not in adipostasis (Michel et al., 2016). Nevertheless, the existence of different leptin paralogs in fish differentially involved in the regulation on energy resources in a species/tissue dependent manner might explain the variety of results.

### Gastrointestinal hormones

#### Ghrelin

Ghrelin is a peptide mainly synthesized in the stomach or its equivalent in stomachless fish species (Kaiya et al., 2008), although its gene expression is also detected in other peripheral locations and in brain (Kojima et al., 1994; Unniappan et al., 2002; Feng et al., 2013). Some studies point to the endocrine cells in the digestive mucosa as the peripheral primary synthesizing site of ghrelin in fish (Jönsson, 2013). Ghrelin is also widely expressed in fish brain, particularly in hypothalamus (Jönsson and Holmgren, 2012; Sánchez-Bretaño et al., 2015b). Ghrelin requires a post-translational acylation (catalyzed by ghrelin O-acyltransferase, GOAT) before binding to its receptor (Yang et al., 2008). Due to tetraploidization experienced by some fish species, two paralog genes of ghrelin receptor and four or eight receptor subtypes are present in some fish (Kaiya et al., 2013). The wide distribution of transcripts of these receptors, and particularly its presence in gastrointestinal tract, liver, brain sensory areas, and pituitary may indicate multiple targets for the regulation of energy balance by this hormone (Jönsson, 2013). Particularly, the GHS-R1a subtype (specifically involved in energy balance, Kaiya et al., 2010) exhibits a dense expression in discrete hypothalamic nucleus, such as the *lateral recessus nucleus*, in support of the orexigenic role of the ghrelinergic system (Sánchez-Bretaño et al., 2015b).

Ghrelin stimulates feeding in all mammalian species studied so far, primarily through increased release of NPY and AgRP (Patton and Mistlberger, 2013). However, this generalized orexigenic effect is not so evident in fish. Ghrelin is an orexigenic peptide in many fishes (Miura et al., 2006; Kaiya et al., 2008; Picha et al., 2009; Kang et al., 2011; Jönsson, 2013; Penney and Volkoff, 2014; Tinoco et al., 2014a). However, this peptide exerts anorexigenic actions in tilapia (Peddu et al., 2009) and both anorexigenic (Jönsson et al., 2010) and orexigenic (Velasco et al., 2016a,b) effects in rainbow trout. The orexigenic action of ghrelin in goldfish is mediated by hypothalamic activation of NPY (Miura et al., 2006) and orexin-A (Miura et al., 2007; Nisembaum et al., 2014b) mRNA via vagus nerve (Matsuda et al., 2006b), as peripheral administration of ghrelin does not modify NPY mRNA expression in goldfish hypothalamus (Nisembaum et al., 2014b). Interestingly, the expression of ghs-r1 ghrelin receptor (Sánchez-Bretaño et al., 2015b) and GOAT (Blanco et al., 2016b) in hypothalamic nuclei that also express orexin-A and NPY in goldfish reinforces the suggested mechanism for the orexigenic action of ghrelin in this teleost. On the other hand, the post-feeding decrease of circulating ghrelin levels (Unniappan et al., 2004) and the preprandial rise of circulating acyl-ghrelin and GOAT (Blanco et al., 2016a) support the role of this peptide as a meal initiator in goldfish. In agreement with this action of ghrelin, the hypothalamic mRNA expression of GH secretagogue receptors (sbGHSR-1a and sbGHSR-1b) was higher in fasted than in fed seabream (Zhang et al., 2008).

In addition to the effects of ghrelin on the feeding regulatory neurons in hypothalamus, some studies suggest that the effects of ghrelin on food intake are also mediated by gastrointestinal motility in the periphery, as in zebrafish (Olsson et al., 2008). However, this is not the rule in fish, since ghrelin does not modify intestinal motility in other teleost (Kitazawa et al., 2012). Localization studies agree with this lack of ghrelin effect on gut motility since ghrelin receptor expressing cells are not present in the muscle layer of gut (Sánchez-Bretaño et al., 2015b).

Feeding and nutritional status are the main factors involved in the regulation of ghrelinergic system in mammals, but this is controversial in fish. Different responses to food deprivation occurred depending on the species, tissue, and duration of food deprivation. Accordingly, different effects are found on ghrelin release, its expression in different central and peripheral tissues, and GOAT activity (Unniappan et al., 2004; Jönsson et al., 2007; Fox et al., 2009; Hevrøy et al., 2011; Blanco et al., 2016b).

The effect of ghrelin treatment on central nutrient sensing systems was assessed in rainbow trout, and glucosensing systems appear to be activated by ghrelin treatment (Polakof et al., 2011c), an effect opposed to that addressed in mammals (Wang et al., 2008). In agreement with the well-known effect in mammals, fatty acid sensing systems are inhibited in rainbow trout by ICV ghrelin treatment (Velasco et al., 2016a,b). These changes agree with the increased mRNA abundance of AgRP/NPY and decreased mRNA abundance of POMC-A1/CART observed simultaneously (Velasco et al., 2016a,b).

#### CCK

CCK is a member of the CCK-gastrin family that exerts a key role in digestive physiology of vertebrates, including fish (Olsson and Holmgren, 2011). Partial and complete mRNA sequences of CCK are available for a number of fish species, and CCK-like-ir is present in gut and nervous system of several teleost species (Jönsson et al., 1987; Micale et al., 2012, 2014; Ji et al., 2015). CCK-like peptides are potent anorexigenic signals in fish (Himick and Peter, 1994; Volkoff et al., 2005; Rubio et al., 2008; MacDonald and Volkoff, 2009; White et al., 2016). Moreover, these hormones are involved in many digestive functions and glucose homeostasis (Rajjo et al., 1988; Aldman and Holmgren, 1995; Einarsson et al., 1997; Polakof et al., 2011a; Tinoco et al., 2014b). CCK-8 may be involved in the seasonal changes of food intake experienced by salmonids (White et al., 2016), and starvation challenge experiments support the anorexigenic action of CCK-8 in fish. The expression levels of CCK mRNA decreased in brain and intestine after starvation in different fish species (Murashita et al., 2006; Feng et al., 2012; Ji et al., 2015), while CCK is released after feeding in intestine (Aldman and Holmgren, 1995). The mechanisms underlying the anorexigenic effect of CCK-like peptides are unknown, but the lack of effect after peripheral administration in channel catfish (Schroeter et al., 2015) suggests that anorexigenic action occur at the central level. Interactions with other feeding regulators, as the hypothalamic expression of amylin, have been described in goldfish (Thavanathan and Volkoff, 2006), but the mechanisms involved appear to be different when comparing peripheral and central administration (Hoskins and Volkoff, 2012). Accordingly, a differential distribution pattern of CCK receptors subtypes is present in goldfish with high expression of CCKAR subtype in the intestine, whereas the CCKBR subtype predominantly expressed in hypothalamus and vagal lobe (Tinoco et al., 2015). Finally, CCK-8 treatment activated glucosensing capacity in hypothalamus and hindbrain of rainbow trout (Polakof et al., 2011a).

#### Other gastrointestinal hormones

Glucagon-like peptide 1 (GLP-1) appears to be anorectic in fish (Silverstein et al., 2001; White et al., 2016). In rainbow trout GLP-1 treatment activated hypothalamic glucosensing systems (Polakof et al., 2011b) in parallel with altered mRNA abundance of several neuropeptides including increased values for CART and POMC-A1 and decreased values for NPY (Polakof et al., 2011b).

Other gastrointestinal peptides also show anorexigenic properties in fish including those peptides belonging to the calcitonin gene-related peptide family, such as the calcitonin gene-related peptide (CGRP), intermedin (or adrenomedulin) and amylin (or islet amyloid polypeptide). These peptides act through a calcitonin receptor-like receptor and show a broad distribution in brain and peripheral tissues in fish (Ogoshi et al., 2003; Martínez-Álvarez et al., 2008). CGRP mRNA is widely expressed in the central and peripheral nervous system, intermedin-ir is present in brain, pituitary and most peripheral tissues, including endocrine pancreas (López et al., 1999). Amylin is present in Brockmann bodies, thus suggesting that once produced in the periphery transported into brain where it has central actions (Westermark et al., 2002). ICV injections of these three peptides in goldfish induced a decrease in food intake (Martínez-Álvarez et al., 2009) mediated at the central level through unknown mechanisms. The presence of CGRP fibers innervating ventromedial hypothalamic nucleus (Batten and Cambre, 1989) support a direct effect on hypothalamus probably in a paracrine or autocrine manner (Martínez-Álvarez et al., 2009).

## Hypothalamic integration of circadian information

### Hypothalamus and peripheral circadian clocks

**Figure 3:**
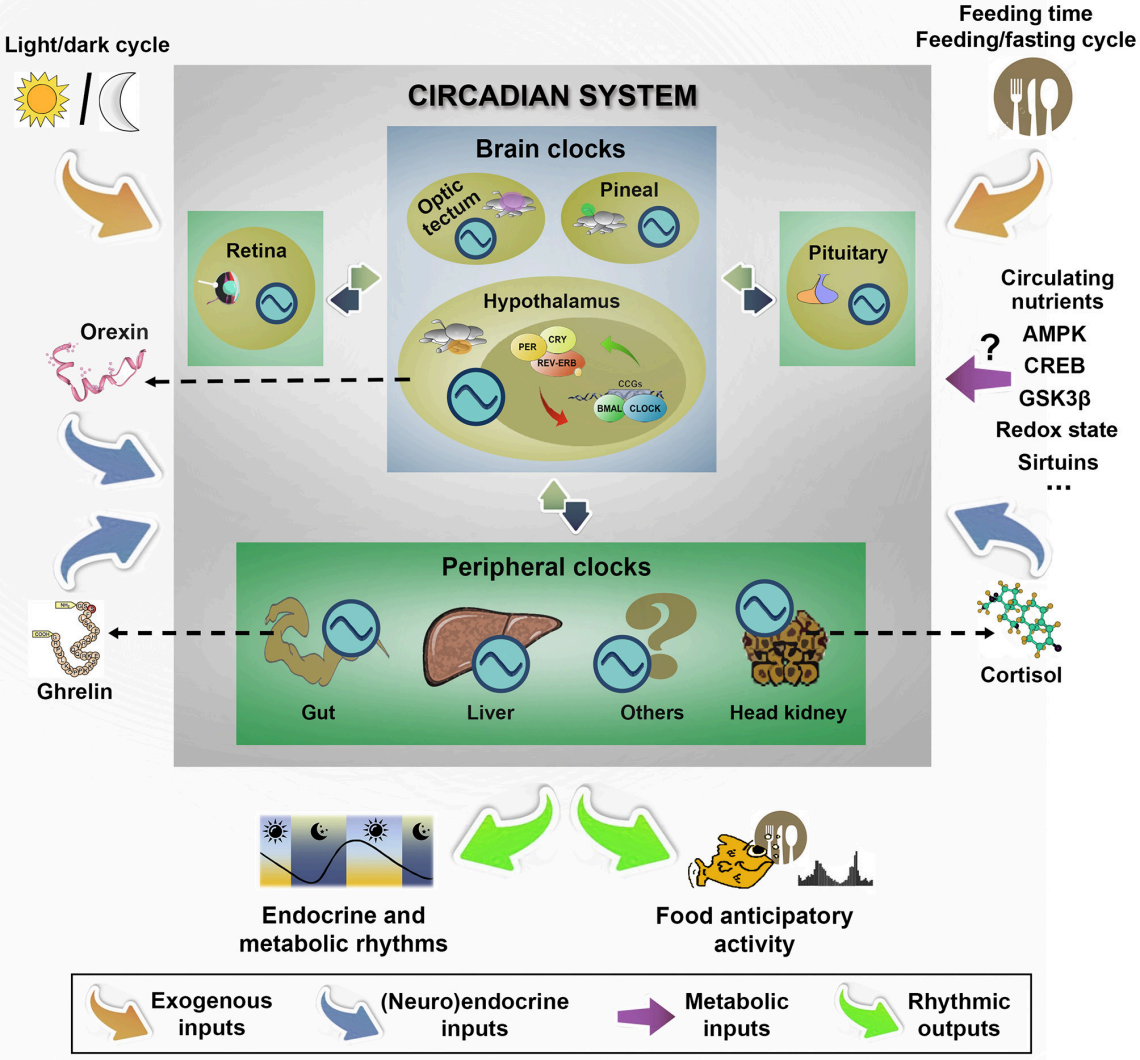
Hallmarks of functional organization of circadian system in fish emphasizing the main external and endogenous inputs that synchronizes brain and peripheral clocks to generate rhythmic outputs, and particularly, the daily food anticipatory activity and food intake. In fish, the lack of a master clock in the functional circadian system organization shapes a more flexible model with different intercommunicated clocks located in many tissues throughout the organism. As external inputs, the light/dark cycle is one of the best characterized, but other time cues (such as feeding time and feeding/fasting cycle) are the dominant exogenous synchronizers of peripheral clocks. The possible role of circulating nutrients and some central and peripheral regulatory signals (such as orexin, ghrelin or cortisol) is discussed in the text. ?, Unknown; AMPK, AMP-activated protein kinase; CREB, cAMP response-element binding protein; GSK3β, Glycogen synthase kinase 3 beta. Clock genes: PER, CRY, REV-ERB, BMAL, CLOCK, CCG. Circadian oscillator: 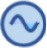.

Feeding shows a rhythmic pattern in a widespread of fish (Madrid et al., 2001), occurring periodically through the 24-h light/dark cycle, but tidal, lunar and seasonal feeding rhythms are also been described in some species (López-Olmeda and Sánchez-Vázquez, 2010). These feeding rhythms, as many other aspects of energy metabolism and homeostatic balance, are coordinated by the phylogenetically well-conserved circadian system, which generates an internal timing that allow animals to anticipate cyclic environmental and endogenous variations, and then, prepare the physiology for upcoming challenges. In the functional organization of circadian system, multiple endogenous clocks, synchronized by environmental and endogenous cues, generate an internal rhythmicity close to 24 h (Albrecht, 2012). The hypothalamus plays a key role in the circadian timekeeping system of vertebrates, acting as an integrative neuronal network. In mammals, a bilateral structure in the anterior hypothalamus, the suprachiasmatic nucleus (SCN), is an autonomously rhythmic nucleus that drives overt circadian rhythms in behavior and physiology, including food intake (Partch et al., 2014). The SCN is formed by clusters of interconnected neurons with individual molecular clock mechanisms inside each cell that function as an autonomous oscillator, and the intercelular coupling among these individual cells is critical for the coordination of endogenous rhythmicity and the outputs to downstream tissues (Evans and Gorman, 2016). Additional neuronal clocks are also present in ARC, ventromedial hypothalamus, and the dorsomedial hypothalamus, and in a variety of peripheral tissues, including liver, adipose tissue, adrenal and muscle (Dibner et al., 2010). In mammals, it is widely accepted that the internal coordination of circadian rhythmicity is established by the temporal signals provided by the master SCN to other hypothalamic nuclei and peripheral organs through a wide variety of direct and indirect pathways that form a network of oscillators. Fish hypothalamus also contains circadian oscillators (Velarde et al., 2009; Patiño et al., 2011; Vatine et al., 2011; Idda et al., 2012; Martín-Robles et al., 2012; Nisembaum et al., 2012; Vera et al., 2013), but no master clock has been yet identified. In contrast, the functional relevance of endogenous clocks in a variety of fish tissues supports the existence of a multiple network of endogenous oscillators in the brain (hypothalamus, diencephalon, pineal, retina, optic tectum, pituitary) and peripheral (liver, gut, gonads, head kidney) locations (Isorna et al., 2017). A summary of the main components in the fish circadian organization is shown in Figure 3.

The key molecules that establish the functional core of self-sustained circadian oscillators clocks are highly conserved from invertebrates to mammals, including fish (Partch et al., 2014), although important differences are found in fish regarding the additional number of copies of the clock genes resulting from the 3R teleost-specific genome duplication. The generation of cellular circadian oscillations is based on autoregulatory 24-h transcriptional/translational feedback loops of a set of clock genes (Ko and Takahashi, 2006). The proteins BMAL1, CLOCK and its analog NPAS2 act as transcriptional activators via E-box sequences driving the expression of clock controlled genes, some of them encoding other core clock protein repressors (PER and CRY). These PER and CRY proteins form complexes that are translocated into the nucleus where they shut down their own expression by removing the CLOCK(NPAS2)/BMAL1 complexes, defining a main negative loop. Some reinforcing loops are formed by the orphan nuclear receptors from the REV-ERBα-β and RORα-β families, to modulate the rhythmic transcription of *Bmal1, Npas* and *Clock* (Guillaumond et al., 2005; Schibler et al., 2015). Recent studies demonstrated that such oscillations in clock genes expression paralleled critical events of chromatin remodeling (Coomans et al., 2015; Masri et al., 2015).

### Feeding as an entrainment cue for the circadian clocks

The light/dark cycle is the most effective signal that entrains the brain circadian clocks (Schibler et al., 2015). However, feeding related cues, including feeding time, nutritional inputs, feeding/fasting cycle and diet composition are the dominant synchronizers of peripheral clocks. Such so-called food-entrained oscillators provide animals the adaptive advantage of anticipate periodic meals within a circadian period of entrainment (Stephan, 2002; Mendoza, 2006). The food-entrained oscillators constitute the basis of the food anticipatory activity, an increase in locomotor activity occurring just before feeding time in anticipation of a predictable daily meal. This activity was evidenced in fish (López-Olmeda and Sánchez-Vázquez, 2010; Feliciano et al., 2011; Vera et al., 2013) and mammals (Mistlberger, 2009), and appears to be independent on hypothalamic SCN in mammals (Stephan, 2002) and hypothalamic clocks in fish (Velarde et al., 2009). To date, the endogenous control of the food anticipatory activity is largely unknown, but recent studies suggest that some orexigenic hormones, such as ghrelin, may be involved both in fish (Nisembaum et al., 2014b) and in mammals (Patton and Mistlberger, 2013).

The location of food-entrained oscillators is not yet certain. Neuronal feeding-synchronized clocks are suggested to reside in ventromedial and dorsomedial hypothalamus (Acosta-Galvan et al., 2011), or even in extrahypothalamic brain areas including *nucleus accumbens* and amygdala in mammals. The search and identification of feeding entrained clocks is a topical subject in fish, and both, peripheral (Nisembaum et al., 2012; Sánchez-Bretaño et al., 2015a) and brain (Feliciano et al., 2011; Vera et al., 2013; Sánchez-Bretaño et al., 2015b) are possible targets. This complex variety of locations of oscillators requires a functional organization among them to generate coordinated responses, and recent data suggest that nutritional cues may act as time givers (Dattolo et al., 2016).

Food intake is a potent phase-resetting agent for peripheral and central clocks, by entraining a number of overt rhythms in animals. The molecular mechanism underlying feeding entrainment remains largely unknown. In mammals, feeding modifies the phase of molecular oscillations in ARC and dorsomedial hypothalamic nuclei independently on the hypothalamic SCN (Feillet et al., 2008). In fish, a daily feeding schedule synchronizes rhythmicity of central (hypothalamus and optic tectum) and peripheral (gut and liver) clock genes (Feliciano et al., 2011; Nisembaum et al., 2012; Vera et al., 2013). The nature of the endogenous signals associated with feeding that may be capable of resetting peripheral clocks is not fully characterized yet, but metabolites, nutrient sensors, hormones, and possibly neuronal signals transmitted from nutrient-sensing areas to peripheral organs are good candidates.

### Metabolic signals and circadian clocks

The circadian system controls the expression of essential genes in numerous metabolic pathways (Ribas-Latre and Eckel-Mahan, 2016), but, in turns, it can be synchronized by its own effectors, generating a bidirectional relationship among the circadian clocks and metabolic signals (Challet, 2013).

Little is known about how specific diet components can modulate the circadian clocks, although it appears that periodic availability of circulating nutrients induces the feeding dependent resetting of peripheral and central circadian networks (Asher and Sassone-Corsi, 2015). In this context, ARC is an important target for such metabolic feedback (Uchida et al., 2016). Certain populations of neurons in ARC show rhythmicity in mammals (Ellis et al., 2008), with circadian rhythms of PER2 *in vitro* (Guilding et al., 2009). The circadian modulation of ARC neurons by circulating metabolites support the timing activation of this center for sensory metabolic information (Van den Top and Spanswick, 2006). The fact that ARC is also able to influence the functionality of the SCN (Yi et al., 2006), suggest that this ARC-SCN reciprocal interaction is essential to maintain a well-balanced metabolic circadian pattern.

Nutrient sensors evolved as time-giving signals by exerting different actions on the circadian clocks. This is the case of AMPK, regulated by glucose and fatty acids, which directly regulates CRYs proteins (Lamia et al., 2009), CREB, glycogen synthase kinase 3 beta, PPARs, and the redox state (Oosterman et al., 2015; Ribas-Latre and Eckel-Mahan, 2016). One interesting link between sensing of cellular energy status and circadian clocks is the control of clock genes expression and deacetylation of clock proteins by the sirtuins (SIRT), a (NAD^+^)-dependent class III of histone deacetylases that are well characterized by their numerous effects on intracellular metabolism. Particularly, the SIRT1 and SIRT6 establish functional links between cellular metabolism and circadian clocks physiology in mammals (Masri et al., 2014; Orozco-Solis et al., 2015). The activity of SIRT1 fluctuates through the feeding/fasting cycle (Cakir et al., 2009) and is involved in the cyclic control of cofactors and peptides of circadian clocks by deacetylating BMAL1 and PER2 in liver (Nakahata et al., 2008) and activates the hypothalamic SCN pacemaker in mice (Chang and Guarente, 2013). The high expression of this enzyme in the ventromedial hypothalamus support the role of this brain area on monitoring metabolic signals through SIRT1, at least in mice. Interestingly, these ventromedial neurons expressing SIRT1 project POMC neurons in the ARC, and innervate the outer shell of SCN, providing a basis to integrate and communicate metabolic signals in the hypothalamus (Orozco-Solis et al., 2015). SIRT6 appears to be involved in the cyclic synthesis of lipids and carbohydrates (Masri et al., 2014), which may reflect differential responses to distinct nutritional intakes. Whether these molecular sensors modulate brain and peripheral clocks in fish remains to be elucidated. Preliminary studies in rainbow trout assessed changes in mRNA abundance of SIRT-1 in parallel with the functioning of fatty acid sensing systems (Velasco et al., 2016a,b). The clearly adaptive significance of circadian clocks entrainment by nutrient status is supported in fishes by the feeding-related rhythms driven by the circadian system in digestive enzymes (Vera et al., 2007; Montoya et al., 2010; Nisembaum et al., 2014b). Furthermore, changes described in parameters related to glucose, fatty acid, and amino acid metabolism in brain (Polakof et al., 2007c) and peripheral tissues (Polakof et al., 2007d; Hernández-Pérez et al., 2015) also support the involvement of nutrient status.

### Endocrine signals in the entrainment of circadian clocks

It is widely accepted that daily endocrine rhythms are outputs of the circadian system, but recent reports suggest that several hormones may act as inputs in the timing signalization of hypothalamic and peripheral clocks (Challet, 2015; Coomans et al., 2015).

Ghrelin may prove to be an endocrine signal that participates in the integration of gastrointestinal signals by the central clocks. The rhythmic expression of ghrelin transcript in hypothalamus, pituitary and gut (Sánchez-Bretaño et al., 2015b), and the periprandial variations in plasma of goldfish (Blanco et al., 2016a), and in hypothalamus of tilapia, zebrafish and goldfish (Peddu et al., 2009; Blanco et al., 2016b) indicate that ghrelin may work as an output of food-entrained oscillators. Ghrelin receptors are widely expressed in fish brain (Chen et al., 2008; Sánchez-Bretaño et al., 2015b), including those hypothalamic areas involved in the integration of food intake control, as well as the preoptic region and the anterior periventricular nucleus (Kaiya et al., 2010; Sánchez-Bretaño et al., 2015b). The expression of clock genes in these hypothalamic areas (Mazurais et al., 2000; Sánchez-Bretaño et al., 2015c) and the induction of *per* genes by ghrelin (Nisembaum et al., 2014b) support the role of ghrelin as an input of food-entrained clocks. *In vivo* (Nisembaum et al., 2014b) and *in vitro* (Sánchez-Bretaño et al., 2016b) studies carried out in goldfish reinforce the role of ghrelin in the feeding and metabolism-related signaling in hypothalamic and liver clocks. Furthermore, some reports indicate that this hormone may also participate in the regulation of food anticipatory activity in goldfish, as it modifies locomotor activity (Matsuda et al., 2006a), and this locomotor activity is blocked by a ghrelin antagonist (Nisembaum et al., 2014b).

The orexin/hypocretin system is involved in the coordination of rhythmic daily functions, such as feeding and energy balance in fish (Matsuda et al., 2012a). Taking in mind the orexigenic role of this regulator and its stimulatory effect on daily activity rhythms in the absence of other inputs (Nisembaum et al., 2014a), the possible role of orexin system in the physiology of certain brain areas involved in food intake and circadian regulation is intriguing. Orexin fibers found in some central oscillators, such as the pineal and the SCN, and other brain regions related to the regulation of activity/rest cycles (Wong et al., 2011; López et al., 2014). As a rule, orexins exert excitatory effects on various brain nuclei with the exception of a direct hyperpolarisation of clock cells in SCN during the night (Belle et al., 2014).

The orexin system is under the control of the molecular clock, as orexin neurons projecting into the pineal show circadian daily rhythmicity driven by the clock (Appelbaum et al., 2010), and daily variations in the hypothalamic expression of orexin in goldfish related to clock genes oscillations (Hoskins and Volkoff, 2012). Nevertheless, recent studies suggest that this peptidergic system also acts as an input to the clockworks. Particularly, this peptide seems to play an important role in food intake anticipation in fish, as its central administration up-regulates *per* genes in hypothalamus (central) and foregut (peripheral) clocks (Nisembaum et al., 2014a). The induction of *per* genes by orexin, as produced ghrelin, questions the existence of synergy between both orexigenic regulators in their actions on the food-entrained clocks.

Glucocorticoids display strong daily rhythms in vertebrates including fish. Cortisol daily rhythms are clear outputs of the circadian system, which are synchronized by the feeding-fasting cycle and feeding time in fish (Isorna et al., 2017). However, little is known about the possible role of glucocorticoids as inputs to the circadian system in fish. Recent reports in goldfish show that cortisol induces *per1a* and *per1b* expression, and represses the positive elements (*bmal1a* and *clock*) in liver clockwork (Sánchez-Bretaño et al., 2016a). Interestingly, such actions of cortisol on the liver, one of the most sensitive peripheral clockwork, link the entrainment of food-entrained clocks by both, metabolic signals and hormones.

## Hypothalamic integration of other factors

### Stress

The endocrine stress response in fish is mediated by both the hypothalamic-sympathetic chromaffin cell and the HPI axes. The activation of both systems restores homeostasis by mobilizing fuel to make energy available to cope with increased metabolic demand (Wenderlar Bonga, 1997; Mommsen et al., 1999). A disruption of the feeding behavior is a common feature of the behavioral response to stress in fish (Bernier and Peter, 2001; Bernier, 2006). The activation of HPI in response to stress involves the synthesis of corticotrophin relasing factor (CRF) in the neurons of the preotic area of the CNS that, in turn, stimulates release of adrenocorticotropic hormone (ACTH) from the corticotrophic cells in the adenohypophysis. ACTH binds to the MC2R in the surface of the interrenal cells of the head kidney to stimulate glucocorticoid release into the blood (Wenderlar Bonga, 1997; Mommsen et al., 1999). Cortisol, the main glucocorticoid in fish mediates many effects of stressors on metabolic and behavioral processes (Barton, 2002; Bernier, 2006; Aluru and Vijayan, 2009).

In the fish brain, CRF is abundantly expressed within the dorsal telencephalic structures but also in the preoptic area and tuberal hypothalamus of zebrafish (Alderman and Bernier, 2007). ICV administration induces a dose-dependent reduction in food intake levels in goldfish (De Pedro et al., 1993; Bernier, 2006) that is reverted by the injection of receptor antagonist (De Pedro et al., 1997; Bernier and Peter, 2001). Goldfish treated with glucocorticoid antagonists or inhibitors of cortisol synthesis, display increased CRF mRNA brain levels and reduced feeding, which again can be reverted by CRF receptor antagonist (Bernier and Peter, 2001). It is suggested that CRF is a major physiological transductor of stress effects on food intake in fish (Bernier, 2006). In addition, CRF seems also to regulate social effects since its increased expression is responsible of the reduced feeding levels in subordinate fish (Doyon et al., 2003).

Recent studies demonstrated that MC4R is able to bind ACTH in the presence of the melanocortin receptor accessory protein 2 in zebrafish. In addition, ACTH administration inhibits food intake in zebrafish but only in animals carrying a functional copy of MC4R (Agulleiro et al., 2013). Together with the ACTH production in carp brain (Metz et al., 1994) this suggests that ACTH can also transduce stress information into feeding circuitry downstream of the melanocortin system. In addition, MC4R is highly expressed in the magnocellular neurons of the preoptic area where CRF is synthesized thus suggesting that central melanocortins could modulate the activity of the CRF neurons (Cerdá-Reverter et al., 2003a; Sánchez et al., 2009). Stress conditions not only regulate CRF and melanocortinergic pathways in fish as the activity of many other central systems vary with stressful conditions and/or increased glucocorticoid signaling. Changes in the activity of central monoaminergic systems including serotoninergic and dopaminergic systems occurred after stressful conditions. These central monoaminergic systems can play a key role in the integration of information during the exposition to the stressor but also in organizing the coordinated stress response (Lanfumey et al., 2008). Studies in rainbow trout demonstrated that the severity of the stressors were able to produce a rated stress response, but serotonin central levels were not accordingly gradated suggesting that some other systems must be integrating the magnitude of the stress response (Gesto et al., 2015). Chronic treatment with fluoxetine (inhibitor of serotonin recapture) is able to reduce whole body cortisol levels and display anxiolytic effects in zebrafish (Egan et al., 2009) but, on the contrary, acute stress induced a rapid increase of serotoninergic and dopaminergic activity in the forebrain of rainbow trout (Gesto et al., 2013). Accordingly, both dopamine (Leal et al., 2009) and serotonin (Ortega et al., 2013) inhibit food intake in fish supporting both dopaminergic and serotoninergic pathways as neuroanatomical substrates for the integration of stress effects on behavioral pathways regulating food intake.

The reduced food intake observed in response to stress in fish could also relate to changes in the ability of nutrient sensing systems to modulate food intake. In rainbow trout chronic stress induced a readjustment in the activity of hypothalamic glucosensing mechanisms (Conde-Sieira et al., 2010a; Otero-Rodiño et al., 2015). The readjustment resulted in an inability of the fish to compensate with changes in food intake those of circulating glucose levels as observed in non-stressed fish. The response of hypothalamic mRNA abundance of CART, POMC, and NPY to glucose changed under stress conditions (Conde-Sieira et al., 2010a; Otero-Rodiño et al., 2015). CRF might be involved in the mechanism through which stress influences food intake control (Evans et al., 2004; McCrimmon et al., 2006). Accordingly, CRF treatment of rainbow trout hypothalamus *in vitro* readjusted glucosensing mechanisms (Conde-Sieira et al., 2011) in a way similar to that observed under stress conditions (Conde-Sieira et al., 2010a).

### Orexins

The orexins/hypocretins are neuropeptides belonging to the incretin gene family of peptides and are involved in many physiological processes in fish, including feeding (Hoskins and Volkoff, 2012), locomotion/rest cycles (Volkoff, 2012; Nakamachi et al., 2014; Nisembaum et al., 2014a) and reproduction (Wong et al., 2011). The cDNA sequences encoding for preproorexin in fish contains orexin A and orexin B, with a high degree of homology with other orexins in vertebrates (Kaslin et al., 2004). Orexins are present in numerous fish species with a broad distribution. Thus, orexin ir-cell bodies, preproorexin mRNA, and orexin proteins are abundant in hypothalamus and preoptic area of most species so far studied (Volkoff, 2015), areas associated with the control of food intake in fish.

Orexins stimulate food intake in all fishes investigated so far. Earlier studies demonstrated this effect in goldfish (Volkoff et al., 1999; Facciolo et al., 2011; Matsuda et al., 2012a), and zebrafish (Novak et al., 2005; Yokobori et al., 2012). Currently, it is generally accepted that orexins are potent orexigenic regulators in fish (Hoskins and Volkoff, 2012; Matsuda et al., 2012a; Penney and Volkoff, 2014). The stimulation of appetite by orexin injections in many of the species studied is consistent with increases in preproorexin brain mRNA expression levels displayed after different periods of food deprivation (Novak et al., 2005; Abott and Volkoff, 2011; Volkoff, 2014). In goldfish, the presence of orexin-like-ir in the hypothalamic *nucleus recessus lateralis* is induced by fasting condition (Nakamachi et al., 2006). These stimulations of the hypothalamic orexin system by fasting suggest that this peptide play a role in long-term regulation of feeding in fish. Thus, short-term periprandial changes in the expression of orexin occurred in fishes. Orexin expression in the brain increases 1 h before the scheduled mealtime in Mexican blind cavefish (Wall and Volkoff, 2013); and hypothalamic preproorexin expression peaks around mealtime, and decreases after feeding in Atlantic cod (Xu and Volkoff, 2007) and orange-spotted grouper (Yan et al., 2011). These results suggest that orexin may be a signal preceding food supply under scheduled feeding conditions. Recently, it has been shown that orexin-A enhances locomotor activity in goldfish (Nakamachi et al., 2014), and synchronizes locomotor activity in goldfish maintained under 24 L and fasting conditions (Nisembaum et al., 2014a), which allowed authors to suggest that orexin might mediate the locomotor activity entrainment by food schedule in this teleost.

Orexin system is a good target to investigate possible interactions among food intake regulators. Central administration of orexin increases the expression of hypothalamic NPY in goldfish (Volkoff and Peter, 2001b; Nisembaum et al., 2014a), and in the orange spotted grouper (Yan et al., 2011). Desensitizing the orexin system by treatment with high doses of orexin A results in a decrease in NPY-induced feeding (Volkoff and Peter, 2001b), while the blocking of NPY receptors reduces feeding induced by orexin A (Kojima et al., 2009). In addition, ICV co-administration of orexin-A and NPY results in a synergistic orexigenic effect in goldfish (Volkoff and Peter, 2001b; Volkoff et al., 2003). Thus, a functional interdependence may exist between orexin-A and NPY in the stimulation of food intake. On the other hand, ICV orexin-A treatment stimulates ghrelin mRNA 1 h post-injection in goldfish foregut. This agrees with the increase of diencephalic ghrelin induced by orexin-A in this teleost (Miura et al., 2007), and demonstrates that the interaction between these two peptides takes place not only into the hypothalamus, but also suggests a brain-gut communication (Unniappan et al., 2004; Nakamachi et al., 2006). Neuroanatomical evidences support the interactions above described among orexin-A and other appetite-regulating peptides, as NPY and ghrelin (Volkoff et al., 2003; Miura et al., 2007). Recent studies suggested a role of tyrosine hydroxylase in the regulation of food intake and locomotor activity in vertebrates (Wall and Volkoff, 2013). The increased mRNA expression levels of this enzyme induced by fasting or orexin in cavefish may suggest the involvement of catecholamines in the actions of orexin (Abott and Volkoff, 2011).

### Melanin-concentrating hormone (MCH)

Melanin-concentrating hormone (MCH) characterized in chum salmon as a circulating-cyclic heptadecapeptide involved in color change (Kawauchi et al., 1983). MCH is mainly produced within the tuberal hypothalamus, stored in the pituitary neural lobe, and released under adaptation to a light-colored background (Cerdá-Reverter and Canosa, 2009). MCH is a potent orexigenic factor inducing weight gain in mammals (Qu et al., 1996). In fish, however, contradictory results were obtained, such as in goldfish (Matsuda et al., 2006c). Data reported in barfin flounder showed increased hypothalamic MCH expression after food deprivation (Takahasi et al., 2004) and enhanced somatic growth after white background adaptation (Yamanome et al., 2005). Transgenic medaka overexpressing MCH exhibit lightened body color, but development, growth, feeding behavior and reproduction do not remarkably differ from non-transgenic siblings (Kinoshita et al., 2001).

## Conclusions

We aimed with this review to show readers the existing information about the way in which fish hypothalamus integrates information of metabolic, endocrine, and circadian nature to elicit a coordinated food intake response. Compared with the mammalian models, the available studies in fish are more limited resulting in the lack of knowledge in several important aspects of hypothalamic integration. Thus, we have almost no idea about how mechanisms governing food intake operate in the long-term since the available information in fish refers to mechanisms operating at short-term periods. The absence of knowledge is also relevant in terms of characterizing the hypothalamic specific nuclei involved in the process of integration of information as well as in the relationship among cells in those nuclei. In this way, almost all available studies in fish assessed the whole hypothalamus, and this may result in differences in the amount and type of neurons assessed. The large diversity of fish species is not sufficiently covered in the available studies, which in some cases (metabolic control) rely on a handful of species. The possible presence and functioning of amino acid sensing systems in fish, as well as the elucidation of signaling pathways linking activity of sensors with the effectors controlling homeostasis are also open questions demanding further research.

Even in the short-term mechanisms comparable to those known in mammals regarding the existence of neurons involved, presence of nutrient sensing systems and modulatory effects of hormones, the specific responses obtained are not identical to those observed in mammals. In some cases, these differences may relate to the limited available studies in fish precluding us to formulate clear hypothesis. Moreover, other factors might be involved, and we can only speculate about some of them. For instance, the activation of fatty acid sensing systems in mammalian hypothalamus relates to the thermogenic capacity of brown adipose tissue (Contreras et al., 2016), i.e., one of the mechanisms involved in thermoregulation that is not present in fish. Accordingly, the reduced energy expenditure in fish may relate to differential food intake regulation as suggested in a recent review (Van de Pol et al., 2017). A second reason might relate to the gene duplication present in actinopterygians resulting in multiple isoforms of neuropeptides, transporters, enzymes, etc. Accordingly, different subfunctions or different functions may attribute to different isoforms in fish, and this might explain some of the differential effects of hormones when comparing fish and mammals, but this has been evaluated only in a few cases. A third reason may relate to the fact that since fish live in a great variety of habitats they also display many species-specific adaptations such as those related to feeding behavior. Fish eating habits (carnivores, omnivores or herbivores) may be responsible of specific differences in gastrointestinal morphology and hormone functions, and this could be responsible (at least in part) of the differences observed when comparing responses among fish species. Finally, many fish species, including several of the models studied such as rainbow trout, are carnivorous, which is in contrast with the mammalian models studied so far (almost all of them omnivorus/herbivorous species). Accordingly, several of the differences in food intake regulation might relate to this fact. However, this is probably not so simple since the scarce data available in carnivorous mammals (Batchelor et al., 2011) also display differential responses compared with carnivorous fish.

The research carried out in the field in recent years provided evidence for several of the mechanisms involved, though certainly more research is needed, especially to ascertain the interactions among different regulatory mechanisms as well as to establish what are the intracellular common mechanisms through which the information is integrated to elicit a food intake response. Ongoing research in our groups, as well as in many others, will provide responses in the near future.

## Author contributions

All authors listed, have made substantial, direct and intellectual contribution to the work, and approved it for publication.

### Conflict of interest statement

The authors declare that the research was conducted in the absence of any commercial or financial relationships that could be construed as a potential conflict of interest.
